# SUMOylation modulates eIF5A activities in both yeast and pancreatic ductal adenocarcinoma cells

**DOI:** 10.1186/s11658-024-00533-5

**Published:** 2024-01-16

**Authors:** Rocío Seoane, Tomás Lama-Díaz, Antonia María Romero, Ahmed El Motiam, Arantxa Martínez-Férriz, Santiago Vidal, Yanis H. Bouzaher, María Blanquer, Rocío M. Tolosa, Juan Castillo Mewa, Manuel S. Rodríguez, Adolfo García-Sastre, Dimitris Xirodimas, James D. Sutherland, Rosa Barrio, Paula Alepuz, Miguel G. Blanco, Rosa Farràs, Carmen Rivas

**Affiliations:** 1 Centro de Investigación en Medicina Molecular (CIMUS), IDIS, Universidade de Santiago de Compostela, Avda Barcelona, 15706 Santiago de Compostela, Spain; 2 Departamento de Bioquímica e Bioloxía Molecular, Universidade de Santiago de Compostela, 15706 Santiago de Compostela, Spain; 3 Departamento de Bioquímica y Biología Molecular, Facultad de Ciencias Biológicas, Universitat de València, Burjassot, 46100 Valencia, Spain; 4 Centro de Investigación Príncipe Felipe, 46012 Valencia, Spain; 5 Research Department in Genomics and Proteomics, Instituto Conmemorativo Gorgas de Estudios de la Salud, 0816-02593 Panamá, Republic of Panama; 6 Laboratoire de Chimie de Coordination LCC-UPR 8241-CNRS, 31400 Toulouse, France; 7 Present Address: Department of Microbiology, Icahn School of Medicine at Mount Sinai, New York, NY USA; 8 Global Health and Emerging Pathogens Institute, Icahn School of Medicine at Mount Sinai, New York, NY 10029 USA; 9 Division of Infectious Diseases, Department of Medicine, Icahn School of Medicine at Mount Sinai, New York, NY USA; 10 The Tisch Cancer Institute, Icahn School of Medicine at Mount Sinai, New York, NY USA; 11 Montpellier Cell Biology Research Center (CRBM), CNRS-UMR 5237 Université de Montpellier, Montpellier, France; 12 Center for Cooperative Research in Biosciences (CIC bioGUNE), Basque Research and Technology Alliance (BRTA), Bizkaia Technology Park, Building 801A, 48160 Derio, Spain; 13 Instituto Bio TecMed, Universitat de València, Burjassot, 46100 Valencia, Spain; 14 Departamento de Biología Molecular y Celular, Centro Nacional de Biotecnología (CNB), CSIC, Darwin 3, 28049 Madrid, Spain; 15 Present Address: Centro Andaluz de Biología Molecular y Medicina Regenerativa (CABIMER), C/ Américo Vespucio 24, Edificio Cabimer, 41092 Seville, Spain; 16 Present Address: Lunenfeld-Tanenbaum Research Institute, Mount Sinai Hospital, Toronto, ON M5G 1X5 Canada

**Keywords:** eIF5A, Pancreatic ductal adenocarcinoma, Stress granules, Stress response, SUMO2

## Abstract

**Background:**

The eukaryotic translation initiation protein eIF5A is a highly conserved and essential factor that plays a critical role in different physiological and pathological processes including stress response and cancer. Different proteomic studies suggest that eIF5A may be a small ubiquitin-like modifier (SUMO) substrate, but whether eIF5A is indeed SUMOylated and how relevant is this modification for eIF5A activities are still unknown.

**Methods:**

SUMOylation was evaluated using in vitro SUMOylation assays, Histidine-tagged proteins purification from His6–SUMO2 transfected cells, and isolation of endogenously SUMOylated proteins using SUMO-binding entities (SUBES). Mutants were engineered by site-directed mutagenesis. Protein stability was measured by a cycloheximide chase assay. Protein localization was determined using immunofluorescence and cellular fractionation assays. The ability of eIF5A1 constructs to complement the growth of *Saccharomyces cerevisiae* strains harboring thermosensitive mutants of a yeast *EIF5A* homolog gene *(HYP2)* was analyzed. The polysome profile and the formation of stress granules in cells expressing Pab1–GFP (a stress granule marker) by immunofluorescence were determined in yeast cells subjected to heat shock. Cell growth and migration of pancreatic ductal adenocarcinoma PANC-1 cells overexpressing different eIF5A1 constructs were evaluated using crystal violet staining and transwell inserts, respectively. Statistical analysis was performed with GraphPad Software, using unpaired Student’s *t*-test, or one-way or two-way analysis of variance (ANOVA).

**Results:**

We found that eIF5A is modified by SUMO2 in vitro, in transfected cells and under endogenous conditions, revealing its physiological relevance. We identified several SUMO sites in eIF5A and found that SUMOylation modulates both the stability and the localization of eIF5A in mammalian cells. Interestingly, the SUMOylation of eIF5A responds to specific stresses, indicating that it is a regulated process. SUMOylation of eIF5A is conserved in yeast, the eIF5A SUMOylation mutants are unable to completely suppress the defects of *HYP2* mutants, and SUMOylation of eIF5A is important for both stress granules formation and disassembly of polysomes induced by heat-shock. Moreover, mutation of the SUMOylation sites in eIF5A abolishes its promigratory and proproliferative activities in PANC-1 cells.

**Conclusions:**

SUMO2 conjugation to eIF5A is a stress-induced response implicated in the adaptation of yeast cells to heat-shock stress and required to promote the growth and migration of pancreatic ductal adenocarcinoma cells.

**Graphical Abstract:**

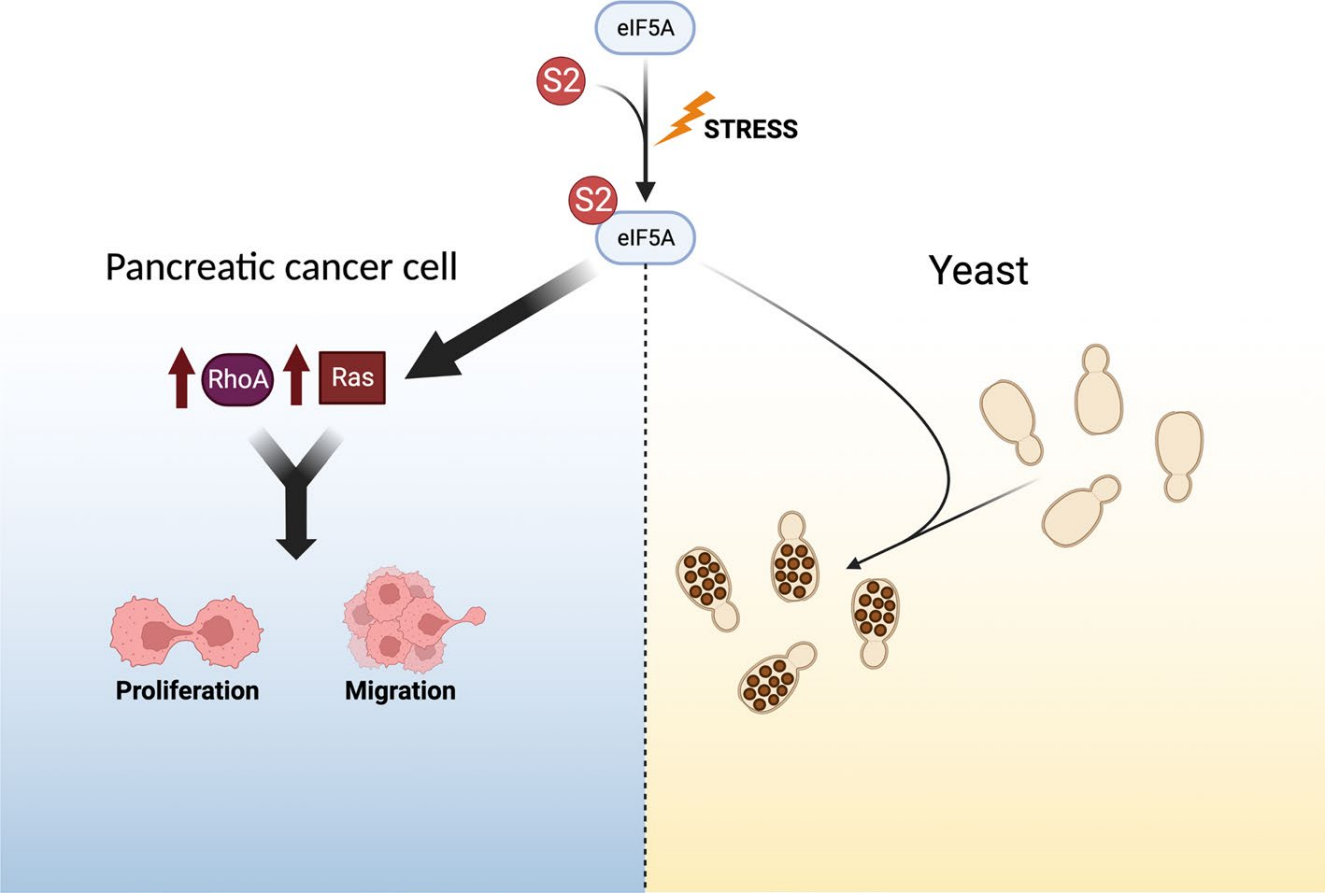

**Supplementary Information:**

The online version contains supplementary material available at 10.1186/s11658-024-00533-5.

## Background

The eukaryotic translation initiation factor 5A (eIF5A) is a highly conserved protein involved in translation elongation, transcription, mRNA turnover, and nucleocytoplasmic transport [1–6], and is implicated in different cellular processes including immune cell differentiation, development, metabolic regulation, aging, and ischemic tolerance [7]. In mammals, there are two eIF5A isoforms, eIF5A1 and its highly related eIF5A2. The two human eIF5A forms share 84% sequence identity. The eIF5A1 isoform is constitutively expressed in all tissues, whereas the eIF5A2 protein shows tissue-specific dependency. The protein level of eIF5A2 has been reported to be too low to be detected in most mammalian cells and tissues [8], but it is highly expressed in many cancers and it has been proposed as an oncogene [8–12]. A correlation between increased levels of eIF5A1 and cancer has also been reported and both isoforms have been proposed as biomarkers in several human cancers, including pancreatic ductal adenocarcinoma [1]. Facilitating resistance of cancer cells to the stress conditions to which they are usually subjected is among the multiple proposed functions of eIF5A. Under adverse conditions, eIF5A is involved in the formation of stress granules (SGs) [2], which are essential structures for the cellular response to stress. Assembly and disassembly of SGs are modulated by different posttranslational modifications including phosphorylation, ubiquitination, poly-ADP ribosylation, arginine methylation, NEDDylation, and SUMOylation [12–18]. eIF5A can also be regulated through different posttranslational modifications [19, 20]. Conjugation of ubiquitin to eIF5A has been shown to modulate its stability and proteasome-mediated degradation [21], and while acetylation inactivates the protein [22], hypusine modification is essential for eIF5A-dependent formation of SGs, eukaryotic cell proliferation [7, 16, 23–25], and *Saccharomyces cerevisiae* viability [26]. Several proteomic studies have also pointed to eIF5A as a putative stress-induced SUMOylation target [27–30]. However, whether eIF5A is modified by small ubiquitin-like modifier (SUMO) and what the relevance of this modification is for eIF5A activities are still unknown.

SUMO conjugation or SUMOylation has arisen as a major posttranslational regulatory process in which one or more SUMO peptides are conjugated to specific lysine residues on target proteins via an enzymatic process [31]. This modification process involves the SUMO-activating enzyme SAE1/SAE2, the SUMO-conjugating enzyme UBC9, and an E3 ligase. SUMOylation is reversible by the action of members of the SUMO-specific protease (SENP) family. In mammals, there are five different SUMO isoforms, SUMO1–5. SUMO1 shares 45% amino acid sequence identity with SUMO2 and SUMO3, while those share 96% identity and they are often referred to as SUMO2/3. SUMO1 and SUMO2/3 are ubiquitously expressed whereas SUMO4 is found in kidney, pancreatic cells, placenta, and immune system [32–34] and SUMO5 is limited to testis and blood cells [35, 36]. The effects of SUMOylation are substrate dependent. At the molecular level, SUMOylation modulates protein–protein or protein–nucleic acid interactions, resulting in altered subcellular localization, activity, or stability. SUMOylation is involved in the regulation of transcription, chromatin structure, DNA damage repair, immune responses, carcinogenesis, cell cycle progression, apoptosis, and the stress response, and a deregulation of SUMOylation is known to contribute to the development of several diseases [30, 37–39].

In this study, we demonstrate that both eIF5A1 and eIF5A2 proteins are modified by SUMO in mammalian cells. SUMOylation of eIF5A1, which does not require hypusination, modulates its subcellular localization and stability and is regulated by stress. Importantly, SUMOylation of eIF5A is conserved in yeast, the SUMOylation mutants of eIF5A1 are unable to completely rescue the eIF5A knockout and show a partially defective disassembly of polysomes and formation of SGs upon by heat-shock stress. Moreover, we show that the proproliferative and promigratory activities of eIF5A on pancreatic ductal adenocarcinoma cells require its SUMOylation. In summary, here we show that eIF5A SUMOylation has an important impact on translation inhibition and SGs formation during stress as well as on the proliferative and migratory capacity of pancreatic cancer cells.

## Materials and methods

### Cells and reagents

Human lung adenocarcinoma A549, human pancreatic carcinoma PANC-1, and human embryonic kidney HEK-293 cells were cultured in Dulbecco’s modified Eagle’s medium (DMEM) supplemented with 10% fetal bovine serum (FBS), 1% l-glutamine (Sigma-Aldrich, Madrid, Spain), and 1% penicillin–streptomycin (Sigma-Aldrich). We used cycloheximide (Sigma-Aldrich) at a final concentration of 100 μg/ml for the indicated times. PANC-1 cells stably expressing pcDNA, pcDNA–HA–eIF5A–WT, pcDNA–HA-eIF5A–3KR, or pcDNA–HA–eIF5A–5KR were generated by transfection and selection with G418 (1500 μg/ml). ML-792 was purchased from MedChemExpress. TAK-243 was purchased from Selleck Chemicals.

### Transfection and plasmids

Cells were transfected using polyethylenimine (Polysciences, Hirschberg an der Bergstrasse, Germany) following the instructions of the manufacturer. pcDNA–His6–SUMO2 and pcDNA–UBC9–SV5 expression plasmids have been previously described [40, 41]. Flag–eIF5A1 and Flag–eIF5A1K50R were kindly provided by Dr. Park (NIDCR, NIH, USA). For yeast expression experiments, eIF5A1 wild type (WT) or mutant coding sequences were subcloned first into a pENTR221 vector with a C-terminal Flag6-TEV2–His10 tag by PCR and then into the 2 µ derivative pAG425GPD–ccdB [42] by Gateway LR Clonase II (ThermoFisher). The plasmids encoding HA-tagged eIF5A (HA–eIF5A) or HA–eIF5AK50R were created by subcloning of the eIF5A or eIF5AK50R coding cDNA into the pcDNA–HA or pCMV–HA plasmids, respectively. Pab1–GFP plasmid was kindly provided by Dr. Roy Parker. We used the QuickChange lightning site-directed kit (Agilent Technologies, Madrid, Spain), with the primers listed in Table 1, and Flag–eIF5A or HA–eIF5A plasmids as a template for the site-directed mutagenesis. All mutations were verified by sequencing.

**Table 1:** Oligonucleotides

Oligonucleotide name	Sequence
eIF5AK27A-F	5′-TGCTCAGCATTACGTGCGAATGGCTTTGTGGTG-3′
eIF5AK27A-R	5′-CACCACAAAGCCATTCGCACGTAATGCTGAGCA-3′
eIF5AK34A-F	5′-GGCTTTGTGGTGCTCGCAGGCCGGCCATGTAAG-3′
eIF5AK34A-R	5′-CTTACATGGCCGGCCTGCGAGCACCACCACAAAGCC-3′
eIF5AK68A-F	5ʹ-ATCTTTACCGGGAAGGCATATGAAGATATCTGC-3ʹ
eIF5AK68A-R	5′-GCAGATATCTTCATATGCCTTCCCGGTAAAGAT-3′
eIF5AK84A-F	5′-GATGTCCCCAACATCGCAAGGAATGACTTCCAG-3′
eIF5AK84A-R	5′-CTGGAAGTCATTCCTTGCGATGTTGGGGACATC-3′
eIF5AK126A-F	5′-GGAGATTGAGCAGGCGTACGACTGTGGAGAAGA-3′
eIF5AK27R-F	5′-TGCTCAGCATTACGTCGGAATGGCTTTGTGGTG-3′
eIF5AK27R-R	5′-CACCACAAAGCCATTCCGACGTAATGCTGAGCA-3′
eIF5AK34R-F	5′-GGCTTTGTGGTGCTCCGAGGCCGGCCATGTAAG-3′
eIF5AK34R-R	5′-CTTACATGGCCGGCCTCGGAGCACCACCACAAAGCC-3′
eIF5AK68R-F	5′-ATCTTTACCGGGAAGCGATATGAAGATATCTGC-3′
eIF5AK68R-R	5′-GCAGATATCTTCATATCGCTTCCCGGTAAAGAT-3′
eIF5AK84R-F	5′-GATGTCCCCAACATCCGAAGGAATGACTTCCAG-3′
eIF5AK84R-R	5′-CTGGAAGTCATTCCTTCGGATGTTGGGGACATC-3′
eIF5AK126R-F	5′-GGAGATTGAGCAGCGGTACGACTGTGGAGAAGA-3′
eIF5AK126R-R	5′-TCTTCTCCACAGTCGTACCGCTGCTCAATCTCC-3′
OTL187	5′-ACCTTAGCGATCGCATGTCTGACGAAGAACATACC-3′
OTL188	5′-ACCTTAACGCGTATCGGTTCTAGCAGCTTCC-3′

### In vitro SUMOylation assay

For the in vitro transcription/translation of proteins we used a rabbit reticulocyte-coupled transcription/translation system (Promega, Madrid, Spain) and ^35^*S*-methionine or unlabeled (cold) methionine. In vitro translated proteins were subjected to in vitro SUMOylation assays using recombinant E1 (Biomol, Lausen, Switzerland), UBC9, and SUMO2, as previously described [43].

### In vitro deSUMOylation assay

We performed an in vitro deSUMOylation assay on eIF5A1–SUMO2 with recombinant SUMO specific peptidase 1 (GST–SENP1) (Biomol) as described previously [44]. eIF5A1–SUMO2 proteins obtained after in vitro SUMOylation assay were incubated with GST–SENP1 in 30 μl of reaction buffer containing 50 mM Tris (pH 7.5), 2 mM MgCl_2_, and 5 mM beta-mercaptoethanol at 37 °C for 1 h. Reactions were terminated by adding sodium dodecyl-sulfate polyacrylamide gel electrophoresis (SDS–PAGE) loading buffer, boiled for 5 min at 100 °C, analyzed by SDS–PAGE, and detected by autoradiography.

### Protein analysis and antibodies

Mammalian cells were scrapped in SDS–PAGE loading buffer and boiled for 5 min at 100 °C. Once the proteins were separated using SDS–PAGE, they were transferred to a 0.45 μm nitrocellulose membrane.

For analysis of the yeast proteins by western blot, cells were grown to exponential phase (OD600 = 0.35–0.8) in SC–Leu at 25 °C. Cultures were then normalized to an OD600 = 0.35 and incubated for an additional 4 h at 37 °C. Cells were then harvested, washed once in 10% trichloroacetic acid (TCA), and disrupted using glass beads in 10% TCA using a Beadbeater. Precipitates were collected by centrifugation, resuspended in 2× SDS-sample buffer, and neutralized with 1 M Tris-Base. Samples were boiled at 99 °C for 5 min, cleared by centrifugation, and separated in 4–20% Tris–glycine gels (Invitrogen, Madrid, Spain).

The primary antibodies used were anti-HA (901513, Biolegend), anti-SUMO2/3 (ab3742, Abcam, Cambridge, UK), anti-eIF5A (ab32443, Abcam), anti-eIF5A2 (17069-1-AP, Proteintech or HPA029090, Sigma-Aldrich), anti-Histidine (MA1-21315, Invitrogen), anti-GAPDH antibody (sc-32233, Santa Cruz Biotechnology), anti-Ras (3965S, Cell Signaling), anti-RhoA mouse (sc179, Santa Cruz Biotechnology), anti-β-actin (sc4778, Santa Cruz Biotechnology), anti-OctA-Probe (sc166355, Santa Cruz Biotechnology), and anti-Flag-M2 HRP-conjugated (A8592, Sigma). Anti-Smt3 antibody was kindly provided by Helle Ulrich (Institut for Molecular Biology, Mainz) [45]. Anti-hypusine antibody (FabHpu24) was kindly provided by Genentech.

### Immunofluorescence

Cells were fixed in 2% paraformaldehyde/PBS and permeabilized with 0.25% Triton X-100/PBS in PBS. Nonspecific binding sites were blocked with 2% BSA/PBS and samples were incubated overnight with primary antibodies. After washing with PBS, coverslips were incubated with Alexa488-conjugated secondary antibody for 1 h at room temperature. Nuclei were stained with 4′,6′-diamidino-2-phenylindole (DAPI), and preparations were mounted with ProLong Diamond antifade Mountant (P36970) and analyzed using a Confocal Leica microscope.

### Nuclear-cytoplasm fractionation

The nuclear and cytoplasmic fractions were isolated using the REAP method as previously reported [46].

### Histidine purification

The purification of His-tagged conjugates was performed using Ni^2+^-NTA agarose beads, as previously described [47].

### Migration assays

Migration was evaluated using Corning transwell inserts with an 8.0 μm porous filter as previously described [48]. Briefly, 1 × 10^5^ cells were resuspended in 200 μl of serum-free medium and added into the upper chamber while 400 μl of medium supplemented with 10% FBS were added to the lower chamber. At 16 h after incubation, cells were fixed with 100% methanol and stained with 0.1% crystal violet. After removing the nonmigrated cells in the upper chamber, we counted the number of migrated cells by microscopy.

### Cell growth assays

Next, 2 × 10^3^ cells were plated in 24-well plates and allowed to grow for the indicated times. Then, cells were fixed in methanol and stained with 0.1% crystal violet. The dye was extracted with acetic acid (10%) and then absorbance at 590 nm was measured using a spectrophotometer.

### Yeast strains and viability assays

All *S. cerevisiae* strains employed are described in Table 2. PAY717, PAY723, and PAY725 strains were as previously described [49]. For viability assays, wild-type or temperature sensitive strains transformed with pAG425GDP–ccdB derivatives encoding various human eIF5A variants were streaked onto SC–Leu plates and incubated at either 25 °C (permissive) or 37 °C (restrictive) for 72 h. They were then imaged in a GelDoc documentation system with a 0.5 s exposure (Bio-Rad).

**Table 2:** Yeast strains

Strain	Genotype	Source
PAY717	*MATa hyp2-3*	[49]
PAY723	*MATa BNI1–HA*	[49]
PAY725	*MATa hyp2-1 BNI1–HA*	[49]
YTL710	*MATa LEU2::pAG425GPD–ccdB*	This study
YTL718	PAY717 *LEU2::pAG425GPD–ccdB*	This study
YTL720	PAY717*LEU2::pAG425GPD–eIF5a–WT–FTH*	This study
YTL722	PAY717 *LEU2::pAG425GPD–eIF5a–K50R–FTH*	This study
YTL724	PAY717 *LEU2::pAG425GPD–eIF5a–3KA–FTH*	This study
YTL726	PAY723 *LEU2::pAG425GPD–ccdB*	This study
YTL734	PAY725 *LEU2::pAG425GPD–ccdB*	This study
YTL736	PAY725 *LEU2::pAG425GPD–eIF5a–WT–FTH*	This study
YTL738	PAY725 *LEU2::pAG425GPD–eIF5a–K50R–FTH*	This study
YTL740	PAY725 *LEU2::pAG425GPD–eIF5a–3KA–FTH*	This study
YTL1393	PAY725 *LEU2::pAG425GPD–ccdB URA3::pRS416–PAB1–GFP*	This study
YTL1396	PAY725 *LEU2::pAG425GPD–eIF5a–WT–FTH URA3::pRS416–PAB1–GFP*	This study
YTL1399	PAY725 *LEU2::pAG425GPD–eIF5a–K50R–FTH URA3::pRS416–PAB1–GFP*	This study
YTL1404	PAY725 *LEU2::pAG425GPD–eIF5a–3KA–FTH URA3::pRS416–PAB1–GFP*	This study
YTL1823	PAY725 *LEU2::pAG425GPD–eIF5a–3KR–FTH*	This study
YTL1826	PAY725 *LEU2::pAG425GPD–eIF5a–5KR–FTH*	This study
YTL1829	PAY717 *LEU2::pAG425GPD–eIF5a–3KR–FTH*	This study
YTL1832	PAY717 *LEU2::pAG425GPD–eIF5a–5KR–FTH*	This study
YTL1857	YTL710 *LEU2::pAG425GPD–ccdbURA3::pRS416–PAB1–GFP*	This study
YTL1861	PAY725 *LEU2::pAG425GPD–eIF5a–5KR–FTH URA3::pRS416–PAB1–GFP*	This study

^*^All strains are haploid BY4741 derivatives (*MATa his3∆1 leu2∆0 met15∆0 ura3∆0*)

### Polysome analysis

Yeast cells with temperature sensitive eIF5A mutant *hyp2-1* were transformed with different alleles of Histidine- and Flag-tagged human eIF5A1 in a pAG425GPD vector or with the empty vector. Cells were grown in SC–Leu medium at 25 °C until early exponential phase and then incubated at 37 °C for 4 h to deplete endogenous eIF5A. Then, cells were subjected to severe heat-shock stress (46 °C, 30 min). Protein yeast extracts and gradient fractionation of extracts to analyze polysome profiles were done as previously described [50].

### Stress granules analysis

Yeast cells with temperature sensitive eIF5A mutant *hyp2-1* carrying the SG marker Pab1 fused to GFP (Pab1–GFP) were transformed with different alleles of Flag-tagged human eIF5A in a pAG425GPD vector. Cells were grown in SC–Leu–Ura supplemented with 100 µg/ml of Adenine at 25 °C until early exponential phase. Then, cells were subjected to a severe heat-shock stress (46 °C, 10 min) and Pab1 localization was assessed using a Leica DM4 microscope. Images were acquired with 100× objective using a Leica DFC7000T camera and Leica Application Suite X.

### Statistical analysis

The analysis was carried out using GraphPad Prism 8 software (GraphPad Software, San Diego, CA, USA). Comparisons between two groups were performed using a Student’s *t*-test. Comparisons between several groups were performed using one- or two-way analysis of variance (ANOVA) tests. *P*-values of less than 0.05 were regarded as significant.

## Results

### eIF5A protein is modified by SUMO2 in vitro and in vivo in an hypusination-independent manner

**Fig. 1:**
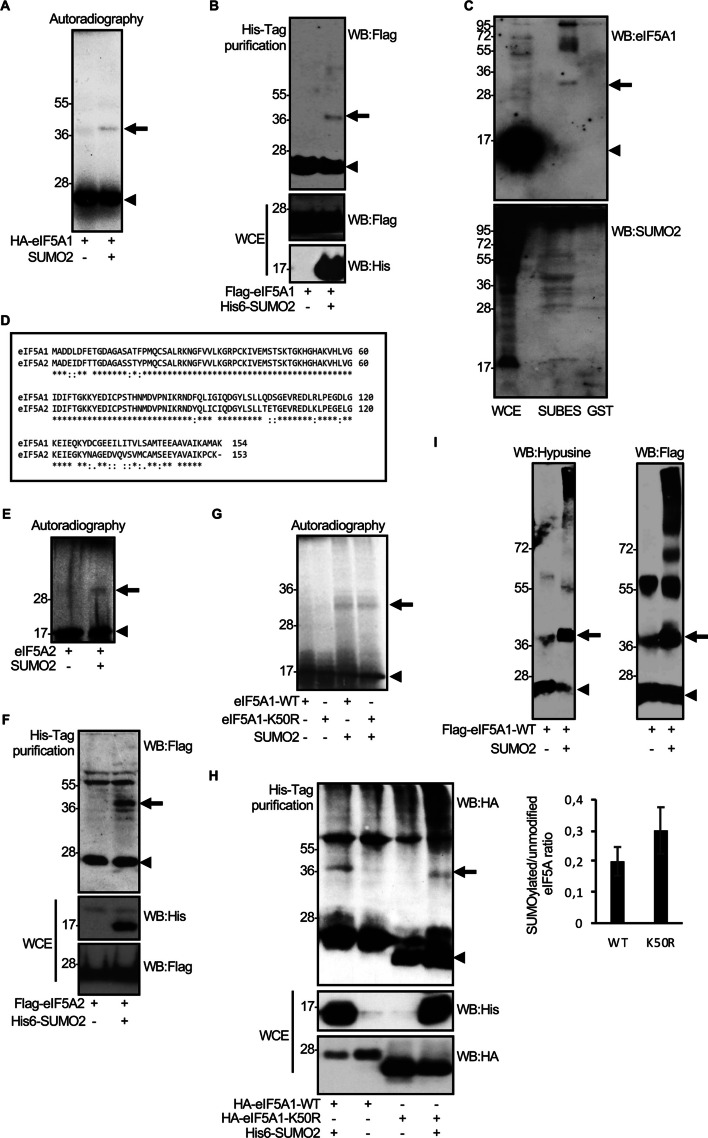
eIF5A1 protein is modified by SUMO2 in vitro and in vivo in an hypusine-independent manner. **A** In vitro SUMOylation assay with SUMO2 using ^35^*S*-methionine labeled in vitro translated HA–eIF5A1 protein. **B** HEK-293 cells were cotransfected with a plasmid encoding Flag–eIF5A1 and pcDNA or Flag–eIF5A1, Ubc9, and His6–SUMO2. Thirty-six hours after transfection, total protein extracts (WCE) and proteins fused to Histidine (His) tag were analyzed by western blot with the indicated antibodies. **C** SUMOylation of endogenous eIF5A1. SUMOylated proteins purified from HEK-293 cells using SUBES were analyzed by western blot with anti-eIF5A1 or anti-SUMO2 antibody. **D** Amino acid sequence homology between human eIF5A1 and eIF5A2. **E** In vitro SUMOylation assay using ^35^*S*-methionine labeled in vitro translated eIF5A2 protein and SUMO2. **F** HEK-293 cells were co-transfected with Flag–eIF5A2 and pcDNA or Flag–eIF5A2, UBC9, and His6–SUMO2. Thirty-six hours after transfection, total protein extracts and proteins fused to Histidine tag were analyzed by western blot with the indicated antibodies. **G** In vitro SUMOylation assay in the presence of SUMO2 and using ^35^*S*-methionine labeled in vitro translated eIF5A1–WT or the eIF5A1–K50R hypusination mutant as a substrate. **H** HEK-293 cells were transfected with HA–eIF5A1–WT or HA–eIF5A1–K50R together with pcDNA or Ubc9 and His6–SUMO2. Thirty-six hours after transfection, whole-protein extracts and Histidine-tagged purified proteins were analyzed by western blot with the indicated antibodies (left panel). The ratio of SUMOylated eIF5A to unmodified eIF5A is shown (right panel). Data represent the mean and error bars of three biological replicates. **I** In vitro translated Flag–eIF5A1–WT protein was subjected to an in vitro SUMOylation assay in the presence of SUMO2 and the SUMOylation products were analyzed by western blot with anti-hypusine or anti-Flag antibodies. Arrowheads in all panels indicate unconjugated eIF5A protein. Arrows indicate the SUMO2-conjugated eIF5A protein

Proteomic data pointed to eIF5A protein as a potential SUMO substrate [27–29]. To verify the SUMOylation of eIF5A, we first evaluated the conjugation of eIF5A1 to SUMO2 in vitro. ^35^*S*-methionine-labeled, in vitro translated human HA–eIF5A1 was subjected to an in vitro SUMOylation assay in the presence of SUMO2. In the absence of SUMO2, HA–eIF5A1 protein was detected as a band of 20 kDa, as expected (Fig. 1A). In the presence of SUMO2, we detected an additional band corresponding to the expected molecular weight of the SUMOylated HA–eIF5A1 protein (Fig. 1A). Similarly, a band of the appropriate size for eIF5A1–SUMO protein, that disappeared after incubation with SENP1, was detected when ^35^*S*-methionine-labeled, in vitro translated untagged eIF5A protein was used as a substrate (Additional file 1: Fig S1). These results indicate that eIF5A1 can be modified by SUMO2 in vitro. To confirm that the protein can be modified also in cells, we evaluated the SUMOylation of Flag–eIF5A1 in HEK-293 cells cotransfected with pcDNA or with pcDNA–UBC9 and pcDNA–His6–SUMO2. Western blot analysis using anti-Flag antibody revealed the appearance of a band of around 20 kDa, corresponding to Flag–eIF5A1, in those cells cotransfected with pcDNA (Fig. 1B). We detected an additional 40 kDa band in those cells cotransfected with His6–SUMO2, indicating that eIF5A1 protein is modified by SUMO2 in these cells (Fig. 1B). Similar experiments carried out using HA–eIF5A1 and His6–SUMO2 confirmed the SUMO2 modification of eIF5A1 in transfected cells (Additional file 1: Fig S2). We then decided to evaluate the SUMOylation of endogenous eIF5A1 protein by using SUMO-binding entities (SUBES), recombinant proteins containing tandem repeats of SUMO interacting motifs (SIMs), thereby allowing purification of SUMO targets under endogenous physiological conditions [51]. Protein extracts from HEK-293 cells were incubated with SUBES or GST and the bound proteins were evaluated by Western-blot analysis with anti-eIF5A1 antibody. As shown in Fig. 1C, we detected the expected band for SUMO conjugated eIF5A1 protein as well as some higher molecular weight bands that may represent additional SUMO-modified forms of eIF5A1 attached to SUBES but not to GST, indicating that endogenous eIF5A1 is SUMOylated in cells without overexpression of the SUMO machinery. Collectively, these results prove that eIF5A1 protein is modified by SUMO2.

The translation factor eIF5A2 shares 84% amino acid homology with eIF5A1 (Fig. 1D). Therefore, we speculated that eIF5A2 may be also regulated by SUMO2. To evaluate this hypothesis, we carried out both an in vitro SUMOylation assay in the presence of SUMO2 using eIF5A2 as a substrate, and a SUMOylation assay with His6–SUMO2 in cells. We observed that eIF5A2 was also modified by SUMO2 in vitro (Fig. 1E) and in cells (Fig. 1F).

eIF5A is the only known protein containing hypusine, an amino acid formed by conjugation of the aminobutyl moiety of spermidine to the lysine residue K50 of eIF5A1 (hypusination). It has been proposed that most eIF5A exists predominantly in its hypusinated form and that this irreversible posttranslational modification is essential for almost all eIF5A activities [52]. To determine whether hypusination modulates SUMOylation, we first analyzed the SUMOylation of eIF5A1 in cells treated or not with the hypusination inhibitor GC7. We observed a small increase in the amount of SUMO2-conjugated eIF5A1–WT protein upon treatment with the hypusination inhibitor GC7 (Additional file 1: Fig S3). We then analyzed the SUMOylation of the hypusination mutant eIF5A1–K50R relative to the WT protein in vitro and in cells. The eIF5A1–K50R mutant was SUMOylated in vitro similarly to the WT protein (Fig. 1G). HA–eIF5A1–K50R was also SUMOylated in cells (Fig. 1H). Quantification of the SUMOylated bands revealed that mutation of the hypusination site slightly increased the amount of SUMOylated protein in comparison with the WT protein but the differences were not significant (Fig. 1H). These results indicated that SUMOylation of eIF5A1 can occur in the absence of hypusination. Finally, we decided to evaluate whether the SUMO2-conjugated eIF5A1 protein could be hypusinated. For that, we carried out an in vitro SUMOylation assay with SUMO2 and using in vitro translated Flag–eIF5A1 protein as a substrate. Then, in vitro SUMOylation products were analyzed by western blot using anti-hypusine or anti-Flag antibody. The anti-hypusine antibody recognized the band corresponding to eIF5A1–SUMO2 protein (Fig. 1I), indicating that eIF5A1 can be both hypusinated and SUMOylated.

### SUMO can conjugate to different residues in eIF5A1

**Fig. 2:**
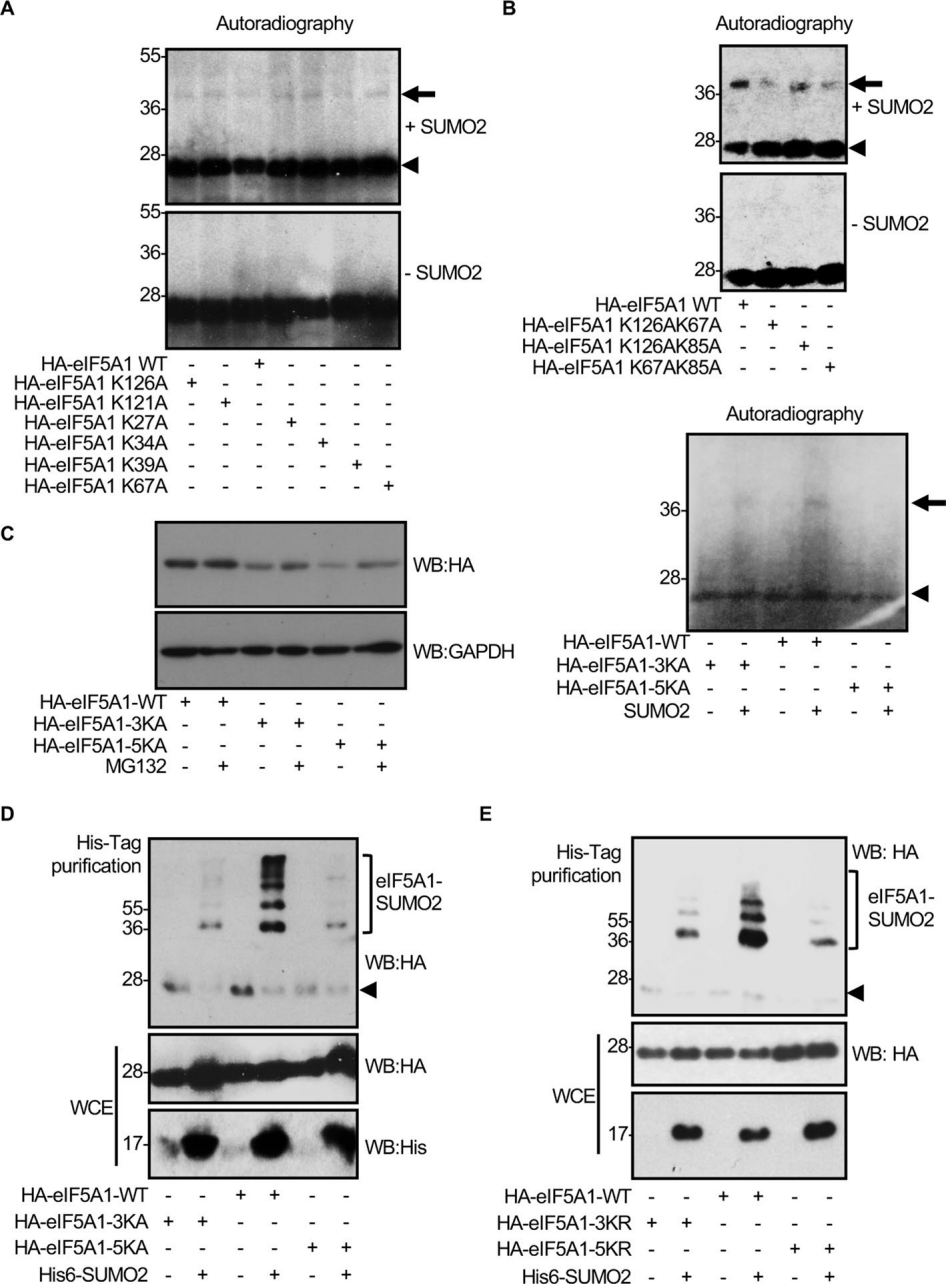
Identification of the SUMO conjugation sites on eIF5A1. **A**, **B** In vitro SUMOylation assay with SUMO2 and the indicated ^35^*S*-methionine-labeled in vitro translated eIF5A1 proteins. **C** HEK-293 cells were transfected with a plasmid encoding HA-tagged eIF5A1–WT, eIF5A1–3KA, or eIF5A1–5KA. Thirty-six hours after transfection, cells were treated with MG132 for 16 h and protein extracts were analyzed by western blot with anti-HA and anti-GAPDH antibodies. **D** HEK-293 cells were transfected with a plasmid encoding HA-tagged eIF5A1–WT, eIF5A1–3KA, or eIF5A1–5KA together with pcDNA or Ubc9 and His6–SUMO2. Thirty-six hours after transfection, cells were treated with MG132 for 16 h and whole-protein extracts (WCE) and Histidine (His)-tagged purified proteins were analyzed by western blot with the indicated antibodies. **E** HEK-293 cells were transfected with a plasmid encoding HA-tagged eIF5A1–WT, eIF5A1–3KR, or eIF5A1–5KR together with pcDNA or Ubc9 and His6–SUMO2. Thirty-six hours after transfection, cells were treated with MG132 for 16 h and whole-protein extracts (WCE) and Histidine (His)-tagged purified proteins were analyzed by western blot with the indicated antibodies. Arrowheads in all panels indicate unconjugated eIF5A1 protein. Arrows indicate the SUMO2-conjugated eIF5A1 protein

In silico analysis of the eIF5A1 amino acid sequence using the web servers GPS-SUMO [53] and JASSA [54] as well as previously reported proteomic data [27–29] suggested that different lysine residues in eIF5A1 such as K27, K34, K39, K67, K68, K85, and K126 can work as SUMO acceptors. Evaluation of single mutants of eIF5A1 in each of these lysine residues did not reveal a reduction in the eIF5A1 SUMOylation (Fig. 2A). We then generated mutants of eIF5A1 with combined mutations and evaluated their SUMOylation in vitro. We observed that mutation of two lysine residues (K126 and K67, K126 and K85, or K67 and K85) reduced the SUMOylation of eIF5A1 (Fig. 2B, upper panel), and that mutation of three (K67, K85, and K126, eIF5A1–3KA mutant) or five lysine residues (K67, K85, K126, K27, and K34, eIF5A1–5KA mutant) led to an even more pronounced reduction in SUMOylation (Fig. 2B, lower panel), indicating that SUMO can conjugate to different residues in eIF5A1 in vitro. To confirm the involvement of the different lysine residues in eIF5A1 SUMOylation in vivo, we decided to evaluate the SUMOylation of the eIF5A1–3KA and eIF5A1–5KA in cells. However, western blot analysis revealed that the levels of the mutant proteins were always lower than that of the WT protein unless we blocked the proteasome, as shown in Fig. 2C. Therefore, we carried out the in vivo SUMOylation assay in presence of the proteasome inhibitor MG132. We observed a clear reduction in the SUMOylation of both mutants in comparison with the WT protein (Fig. 2D). To further verify the specificity of SUMOylation at the different lysine residues, we also generated mutants of eIF5A where lysine residues were replaced by arginine, and evaluated their SUMOylation in cells. We observed that SUMOylation of eIF5A1–3KR and eIF5A1–5KR was reduced in comparison with the WT protein (Fig. 2E), confirming that different lysine residues in eIF5A1 can conjugate SUMO.

### Mutation of the SUMOylation sites in eIF5A alters its stability and subcellular localization in mammalian cells

**Fig. 3:**
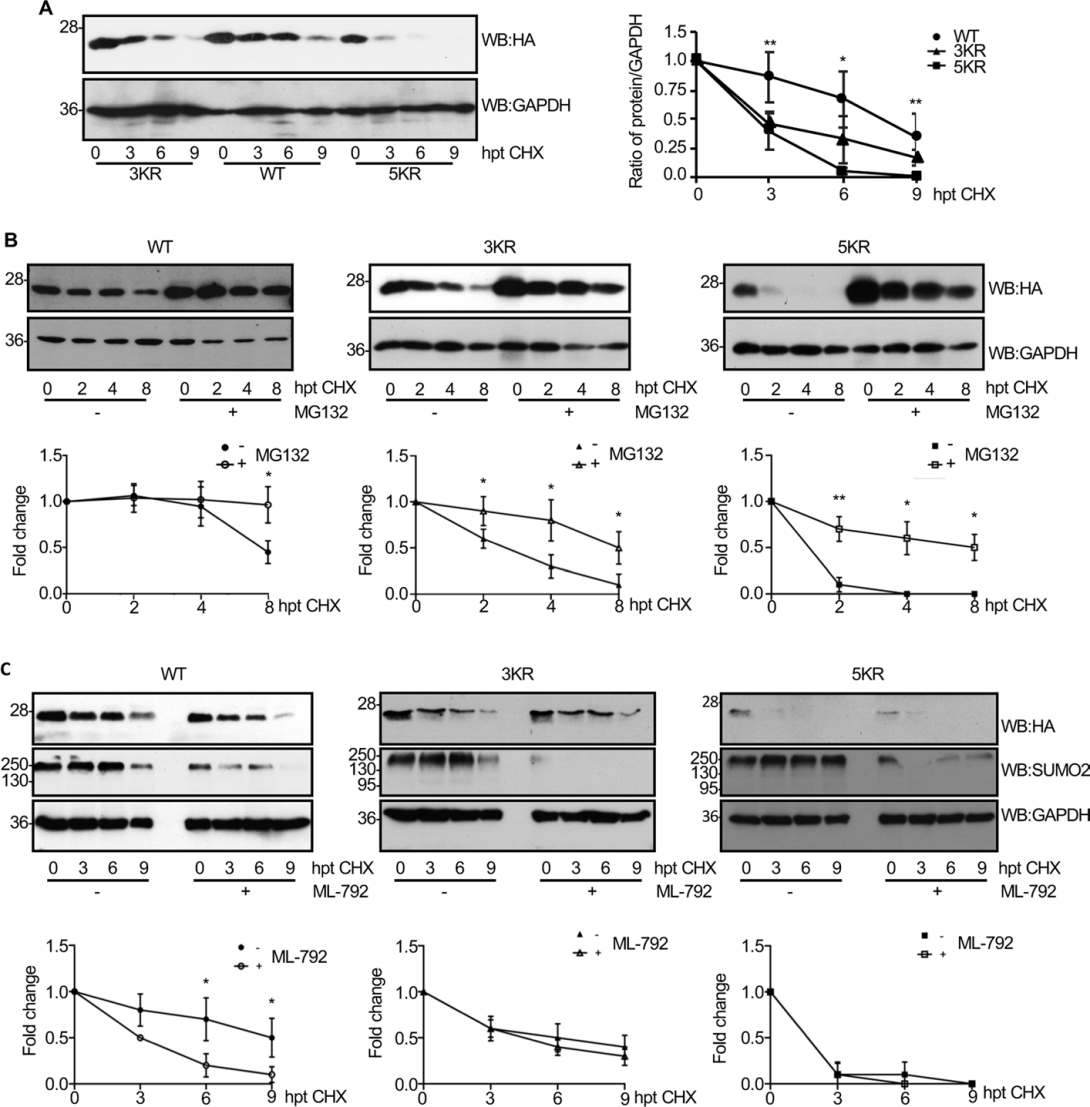
Mutation of the SUMOylation sites in eIF5A1 modulates its stability. **A** HEK-293 cells were transfected with HA–eIF5A1–WT, HA–eIF5A1–3KR, or HA–eIF5A1–5KR, and treated with cycloheximide (CHX) 24 h after transfection. Samples were collected at the indicated hours post treatment (hpt) and protein extracts were analyzed by western blot with the indicated antibodies (left panel). The intensity of the bands was quantified using ImageJ software. eIF5A band intensities were normalized to GAPDH bands from each respective time and plotted (right panel). Data represent the mean and error bars of three biological replicates. Statistical analysis was assessed by a Student’s *t*-test. *P < 0.05, **P < 0.01, compared with WT. **B** HEK-293 cells were transfected and treated as indicated in A but in presence or absence of MG132 treatment. At the indicated times after CHX treatment, protein extracts were analyzed by western blot with the indicated antibodies (upper panels). The intensity of the bands was quantified using ImageJ software. eIF5A band intensities were normalized to actin bands from each respective time and plotted (lower panels). Data represent the mean and error bars of three biological replicates. Statistical analysis was assessed by a Student’s *t*-test. *P < 0.05, **P < 0.01. **C** HEK-293 cells were transfected with HA–eIF5A1–WT, HA–eIF5A1–5KR, or HA–eIF5A–3KR, and 24 h after transfection cells were treated with DMSO or ML-792 (500 nM) for 16 h. Then, the cells were treated with cycloheximide (CHX) for the indicated times and protein extracts were analyzed by western blot with anti-HA antibody (upper panels). The intensity of the bands was quantified using ImageJ software. eIF5A band intensities were normalized to GAPDH bands from each respective time and plotted (lower panels). Data represent the mean and error bars of three biological replicates. Statistical analysis was assessed by a Student’s *t*-test. *P < 0.05

SUMO conjugation may regulate different properties of the substrate including subcellular localization, stability, or activity [31]. As mentioned before, protein levels of eIF5A1–3KA or eIF5A1–5KA mutants were lower than those of WT. Therefore, we decided to evaluate the stability of the SUMOylation mutants. HEK-293 cells transfected with HA–eIF5A1–WT, HA–eIF5A1–3KR/3KA, or HA–eIF5A1–5KR/5KA were treated with cycloheximide, and at different times after treatment, HA–eIF5A1 protein levels were analyzed by western blot using anti-HA antibody, quantified, and plotted. The stability of eIF5A1–3KR/3KA or eIF5A1–5KR/5KA was significantly reduced compared with the stability of the WT protein (Fig. 3A and Additional file 1: Fig S4), suggesting that conjugation of SUMO to eIF5A1 contributes to its stability. Ubiquitination-dependent degradation of eIF5A1 protein has been previously reported [21]. After confirming that the mutation of the lysine residues in eIF5A1 that work as SUMO acceptors did not prevent the conjugation of ubiquitin to eIF5A1 (Additional file 1: Fig S5), we decided to evaluate whether the eIF5A1 SUMOylation mutants were degraded by the proteasome by carrying out stability assays in presence or absence of MG132. The results confirmed the reduced stability of the SUMOylation mutants (Fig. 3B and Additional file 1: Fig S6). In addition, we observed that treatment with MG132 increased the stability of WT and SUMOylation mutants (Fig. 3B and Additional file 1: Fig S6), indicating that mutation of the SUMOylation sites in eIF5A1 promoted its proteasome-mediated degradation. Finally, we decided to evaluate the effect of inhibiting global SUMOylation on the stability of eIF5A1. Treatment with the SUMOylation inhibitor ML-792 reduced the stability of eIF5A1–WT protein but it did not affect to the stability of the SUMOylation mutants (Fig. 3C).

**Fig. 4:**
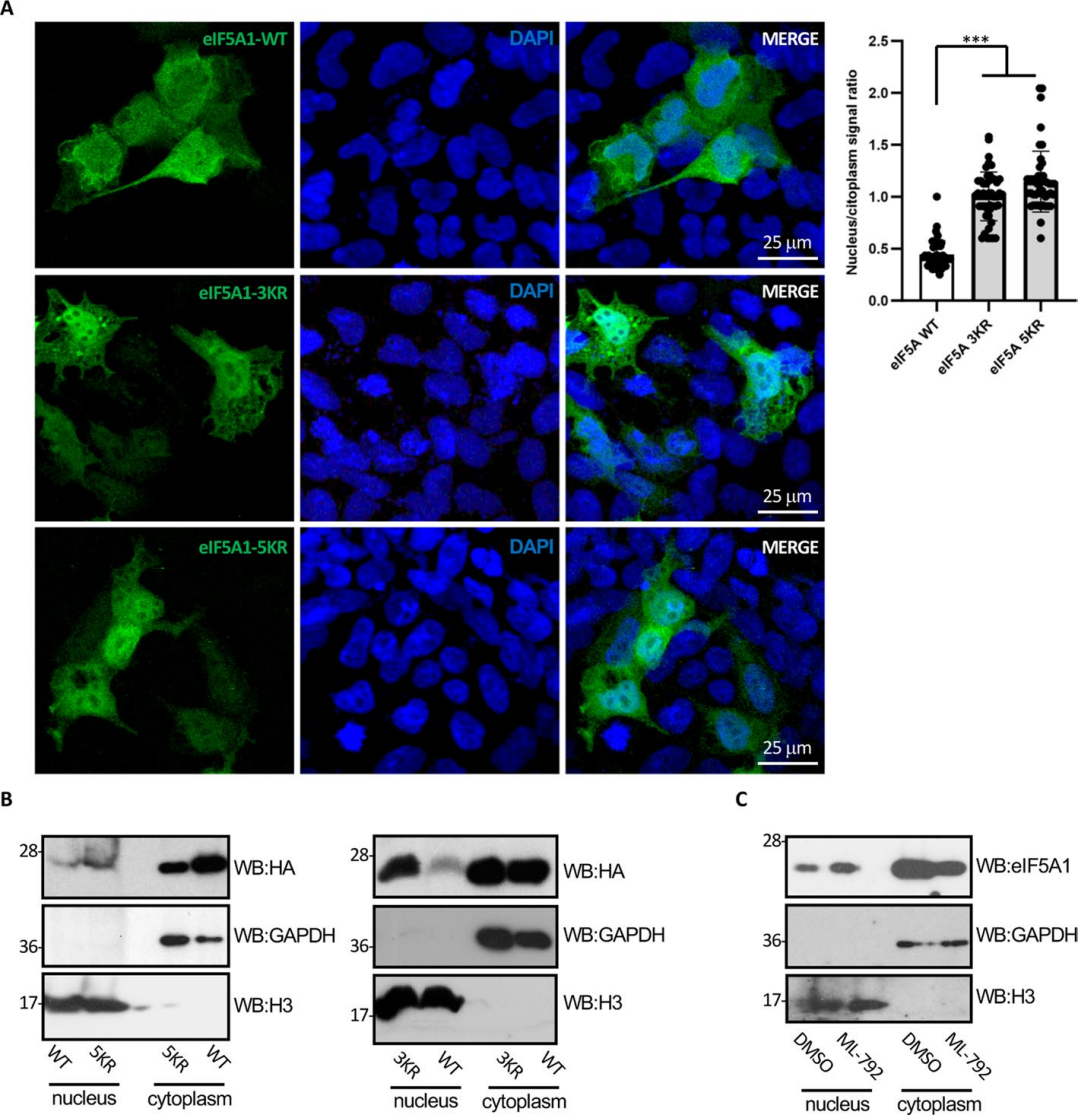
Mutation of the SUMOylation sites in eIF5A1 modulates its subcellular localization. **A** U2Os cells were transfected with HA–eIF5A1–WT, HA–eIF5A1–3KR, or HA–eIF5A1–5KR and 36 h after transfection localization of eIF5A was evaluated using immunofluorescence assay with anti-HA antibody. The nuclear to cytoplasmic ratio was analyzed from images using ImageJ analysis software. Data represent the mean and error bars of 50 cells. Statistical analysis was assessed by a Student’s *t*-test.***P < 0.001. **B** HEK-293 cells were transfected with HA–eIF5A1–WT, HA–eIF5A1–3KR, or HA–eIF5A–5KR, as indicated. Thirty-six hours after transfection, cells were subjected to subcellular fractionation and the levels of eIF5A1 protein in the nucleus and cytoplasm were evaluated using western blot analysis with anti-HA antibody. **C** HEK-293 cells treated with DMSO or the SUMOylation inhibitor ML-792 were subjected to subcellular fractionation and then the levels of the eIF5A1 protein in the nucleus and cytoplasm were evaluated using western blot analysis with anti-eIF5A1 antibody

We then decided to evaluate whether SUMO also modulates the subcellular localization of eIF5A1 protein. U2OS or A549 cells were transfected with HA–eIF5A1–WT, HA–eIF5A1–3KR/3KA, or HA–eIF5A1–5KR/5KA plasmids and 36 h after transfection, we carried out an immunofluorescence staining using anti-HA antibody. Consistent with previous reports, we found that eIF5A1–WT is localized both at the nucleus and cytoplasm of the transfected cells (Fig. 4A) [55]. All the eIF5A1 mutants displayed a more prominent nuclear localization (Fig. 4A and Additional file 1: Fig S7), suggesting that SUMO conjugation favors the cytoplasmic localization of eIF5A1 protein. To test this hypothesis, we carried out subcellular fractionation assays. We observed that the SUMOylation mutants displayed a more nuclear localization than the WT protein (Fig. 4B and Additional file 1: Fig S7). Finally, we studied the effect of inhibiting global SUMOylation on the subcellular localization of eIF5A1. Treatment with the SUMOylation inhibitor ML-792 increased the nuclear localization of eIF5A1 (Fig. 4C). Altogether these results indicated that mutation of the lysine residues susceptible to SUMOylation in eIF5A1 decreases its stability and promotes its nuclear localization.

### SUMOylation of eIF5A1 is modulated by stress

**Fig. 5:**
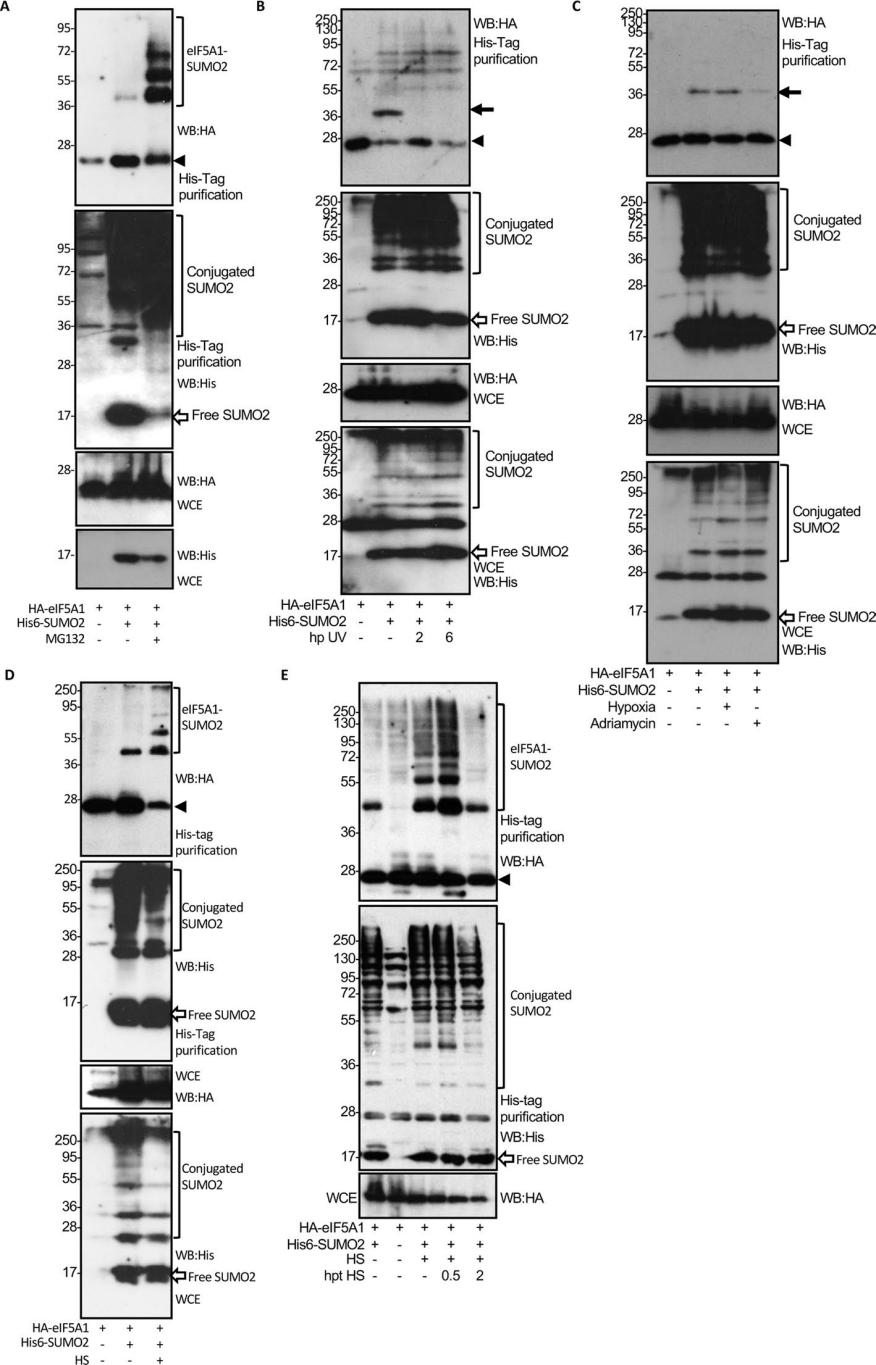
Modulation of eIF5A1 SUMO2 modification upon stress. **A** HEK-293 cells were transfected with a plasmid encoding HA-tagged eIF5A1–WT together with pcDNA or Ubc9 and His6–SUMO2. Thirty-six hours after transfection, cells were treated with DMSO or MG132 for 16 h and whole-protein extracts (WCE) and Histidine (His)-tagged purified proteins were analyzed by western blot with the indicated antibodies. **B** HEK-293 cells were transfected with HA–eIF5A1–WT together with pcDNA or Ubc9 and His6–SUMO2. Thirty-six hours after transfection, cells were subjected to UV irradiation (20 J/m^2^) followed by 2 h or 6 h rest, and then whole-protein extracts and Histidine-tagged purified proteins were analyzed by western blot with the indicated antibodies. Arrow indicates eIF5A–SUMO2 protein. **C** HEK-293 cells were transfected with a plasmid encoding HA-tagged eIF5A1–WT together with pcDNA or Ubc9 and His6–SUMO2. Thirty-six hours after transfection, cells were incubated in hypoxic conditions (1% O_2_) for 8 h or treated with adriamycin 1 μM for 4 h and then whole-protein extracts and Histidine-tagged purified proteins were analyzed by western blot with the indicated antibodies. Arrow indicates eIF5A1–SUMO2 protein. **D** HEK-293 cells were transfected with a plasmid encoding HA-tagged eIF5A1–WT together with pcDNA or Ubc9 and His6–SUMO2. Thirty-six hours after transfection, cells were incubated at 43 °C for 2 h and then whole-protein extracts and Histidine-tagged purified proteins were analyzed by western blot with the indicated antibodies. **E** HEK-293 cells were transfected with a plasmid encoding HA-tagged eIF5A1–WT together with pcDNA or Ubc9 and His6–SUMO2. Thirty-six hours after transfection, cells were first incubated at 43 °C for 2 h (HS) and then incubated at 37 °C for 0.5 or 2 h (hpt HS), as indicated. Then, whole-protein extracts and Histidine-tagged purified proteins were analyzed by western blot with the indicated antibodies. Arrowheads in all panels indicate unconjugated eIF5A1 protein

One of the proposed functions of eIF5A is to regulate translation upon stress, a stimulus that causes a global increase in SUMOylation. To note, analysis of the SUMOylation of eIF5A1 in cells treated with the proteasome inhibitor revealed the appearance of several eIF5A1–SUMO2 bands instead of the unique main eIF5A–SUMO2 band detected in untreated cells (compare Fig. 1B and Fig. 2D), suggesting that proteotoxic stress induced by proteasome inhibition may trigger eIF5A1 SUMOylation. To evaluate this hypothesis, we first analyzed the SUMOylation of eIF5A1–WT in cells treated or not with MG132. We confirmed the appearance of multiple eIF5A1–SUMO2 bands and an increase in the levels of SUMOylated eIF5A1 protein upon treatment with MG132 (Fig. 5A). We then decided to evaluate whether the induction of SUMOylation of eIF5A1 depends on the specific stimulus. We analyzed the SUMOylation of eIF5A1 upon heat-shock stress, UV light irradiation, adriamycin treatment, or hypoxia. We detected a decrease in the SUMOylation of eIF5A1 after irradiation with UV light (Fig. 5B) or adriamycin treatment (Fig. 5C) but we did not observe a change in eIF5A SUMOylation upon hypoxic conditions (Fig. 5C). Finally, we observed that heat stress induced a strong increase in the SUMOylation of eIF5A1 (Fig. 5D), which returns to baseline levels within 2 h after heat shock (Fig. 5E). Altogether, these results indicated that SUMOylation of eIF5A1 depends on the specific stress conditions.

**Fig. 6:**
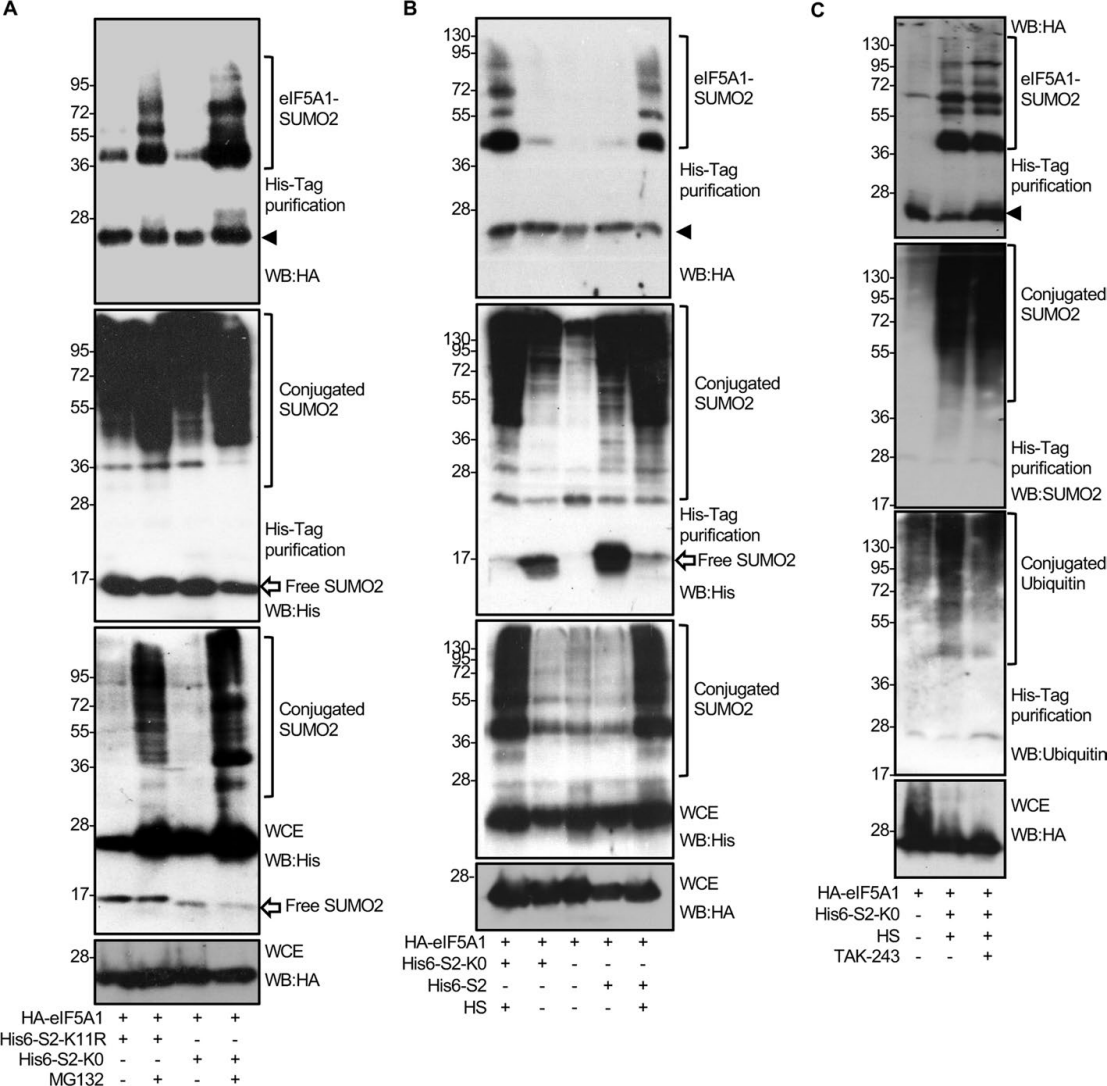
Upregulation of eIF5A1 SUMO2 modification upon stress. **A** HEK-293 cells were cotransfected with HA–eIF5A1–WT, Ubc9, and His6–SUMO2–K11RK48R or His6–SUMO2–K0. 36 h after transfection cells were treated with DMSO or MG132 for 16 h and whole-protein extracts and Histidine-tagged purified proteins were analyzed by western blot with the indicated antibodies. **B** HEK-293 cells were cotransfected with HA–eIF5A1–WT, pcDNA, Ubc9, and His6–SUMO2 or Ubc9 and His6–SUMO2–K0. Thirty-six hours after transfection, cells were incubated at 43 °C for 2 h, as indicated, and whole-protein extracts and Histidine-tagged purified proteins were analyzed by western blot with the indicated antibodies. **C** HEK-293 cells were cotransfected with HA–eIF5A1–WT and pcDNA or Ubc9 and His6–SUMO2–K0. Twenty-four hours after transfection, cells were treated with DMSO or TAK-243, as indicated, and at 12 h after treatment, cells were incubated at 43 °C for 2 h. Whole-protein extracts and Histidine-tagged purified proteins were analyzed by western blot with the indicated antibodies

The accumulation of SUMO2 chains under stress conditions could be explained by the formation of mixed SUMO2–ubiquitin chains. To evaluate this possibility, we analyzed the effect of MG132 treatment on the conjugation of eIF5A1 with a mutant of SUMO2 in lysine residues K11 (SUMO2–K11R) or with a mutant of SUMO2 with no lysine residues (SUMO2–K0). We observed a clear increase in the SUMOylation of eIF5A1 and the appearance of several eIF5A1–SUMO2 bands after inhibition of the proteasome that was independent of the mutation of the lysine residues in SUMO2 (Fig. 6A and Additional file 1: Fig S8). Similar results were observed after evaluating the SUMOylation of eIF5A1 upon heat-shock stress in cells transfected with His6–SUMO2 or His6–SUMO2–K0 (Fig. 6B) or in cells transfected with His6–SUMO2–K0 and treated with the ubiquitination inhibitor TAK-243 (Fig. 6C). Altogether, these results suggest that treatment with MG132 or heat shock induce the multi-mono-SUMOylation of eIF5A1, although we cannot discard the involvement of other ubiquitin-like proteins in the formation of chains on eIF5A.

### Mutation of the SUMOylation sites in eIF5A1 reduces its ability to restore growth of yeast cells with endogenous eIF5A depletion

**Fig. 7:**
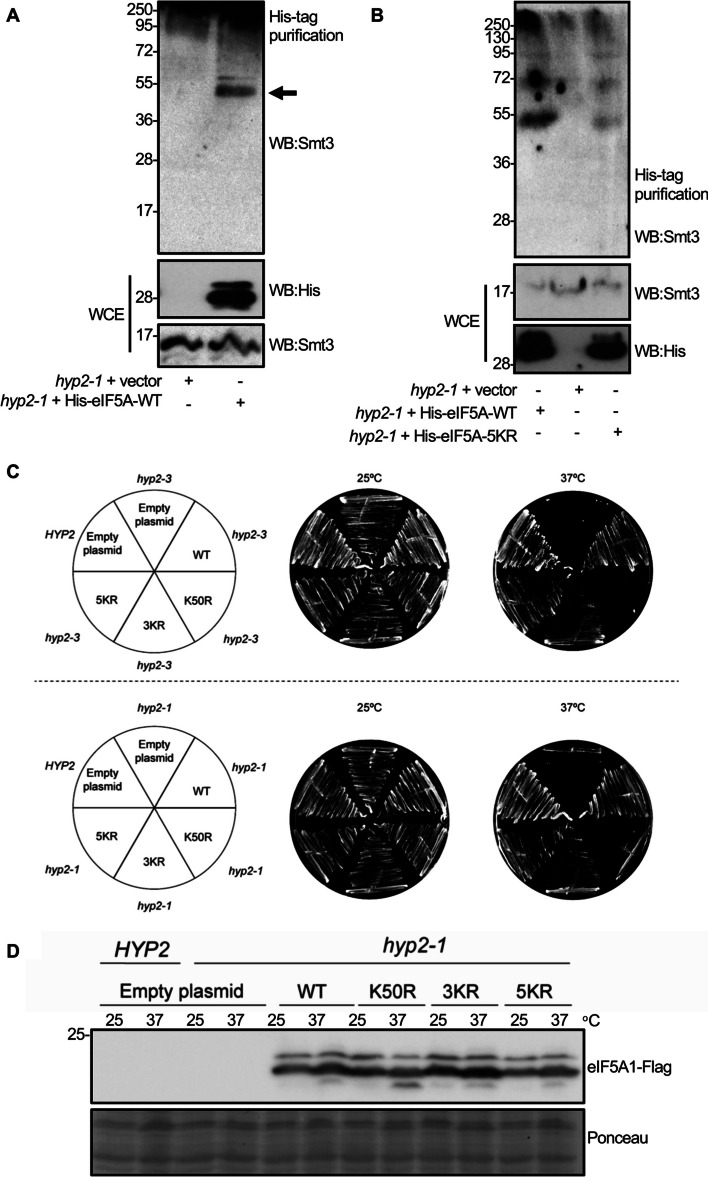
An eIF5A1 SUMOylation-deficient mutant cannot completely substitute for yeast *HYP2*. **A** Histidine-tagged proteins were purified from yeast cells transformed with an empty vector or with Histidine- and Flag-tagged human eIF5A1–WT under denaturing conditions. Then, purified proteins were analyzed by western blot using anti-Smt3 antibody. Arrow indicates Smt3-conjugated eIF5A1 protein. **B** Histidine-tagged proteins were purified from yeast cells transformed with an empty vector, Histidine- and Flag-tagged human eIF5A1–WT or Histidine- and Flag-tagged human eIF5A1–5KR under denaturing conditions. Purified proteins were then analyzed by western blot using anti-Smt3 antibody. **C** WT and *hyp2-1* (upper row) or *hyp2-3* (lower row) yeast strains were transformed with different alleles of Histidine- and Flag-tagged human eIF5A1 in a pAG425GPD vector or with the empty vector. The resulting strains were streaked on SC–Leu plates, incubated at 25 °C or 37 °C for 3 days and imaged in a GelDoc documentation system. **D** Expression levels of the different versions of eIF5A in the indicated strains, growing at 25 °C or after 4 h at 37 °C, were analyzed by western blot. Ponceau staining of the membrane is shown as a loading control

To assess the functionality of the SUMOylation-deficient eIF5A allele, we took advantage of the evolutionary conservation of eIF5A, the essential role of eIF5A for the survival of *S. cerevisiae*, and of the well-established techniques to evaluate the eIF5A function in this model organism. In *S. cerevisiae*, the eIF5A protein is encoded by the two paralogs *HYP1* and *HYP2* [26]. Since *HYP1* is only expressed under anaerobic conditions, *HYP2* is essential for normal aerobic growth [56]. Remarkably, human eIF5A1 can substitute for *HYP2* function and thus restore viability of *hyp2* temperature-sensitive mutants [57]. Therefore, we employed this system to assess whether our SUMOylation-deficient mutant could similarly restore the viability of the *hyp2-3* (C39Y, G118D) and *hyp2-1* (P83S) thermosensitive mutant strains [58, 59] at restrictive temperatures. First, we analyzed the potential SUMOylation of human eIF5A1 in yeast. For that, yeast cells containing an empty vector (*hyp2-1* + vector) or yeast cells with histidine- and Flag-tagged human eIF5A1–WT (*hyp2-1* + His− eIF5A1–WT) were grown at 25 °C and Histidine-tagged proteins were purified under denaturing conditions. Western blot analysis of purified proteins using antibody against the yeast SUMO homolog Smt3 revealed the presence of a band of around 40 kDa, likely corresponding with SUMO-modified eIF5A1 (Fig. 7A), indicating that human eIF5A1 protein is modified by SUMO in yeast, and suggesting that SUMOylation of eIF5A is a highly conserved modification. Similar analysis carried out with yeast cells containing Histidine-tagged eIF5A1–WT or mutants revealed a reduced SUMOylation of the mutants relative to the WT protein (Fig. 7B and Additional file 1: Fig S9). We then transformed yeast *hyp2-3* and *hyp2-1* mutant strains with high-copy plasmids encoding either the wild-type, hypusination-deficient (K50R, as a negative control), or SUMOylation-deficient alleles under the control of the constitutive GPD promoter, and assessed their viability at 37 °C. While both the wild-type strain and the *hyp2* mutant strains bearing all different versions of eIF5A1 exhibited normal growth at the permissive temperature (25 °C), the proliferation of the *hyp2* strains harbouring the SUMOylation mutants was reduced in comparison with the eIF5A1-WT-harbouring *hyp2* thermosensitive strains at the restrictive temperature (Fig. 7C and Additional file 1: Fig S9). This indicates that the SUMOylation-deficient eIF5A1 alleles cannot completely complement *HYP2* function in yeast, despite being expressed at protein levels comparable to the wild-type eIF5A (Fig. 7D and Additional file 1: Fig S9).

### SUMOylation of eIF5A1 is important for polysome disassembly and stress granule formation upon heat-shock stress in *S. cerevisiae*

**Fig. 8:**
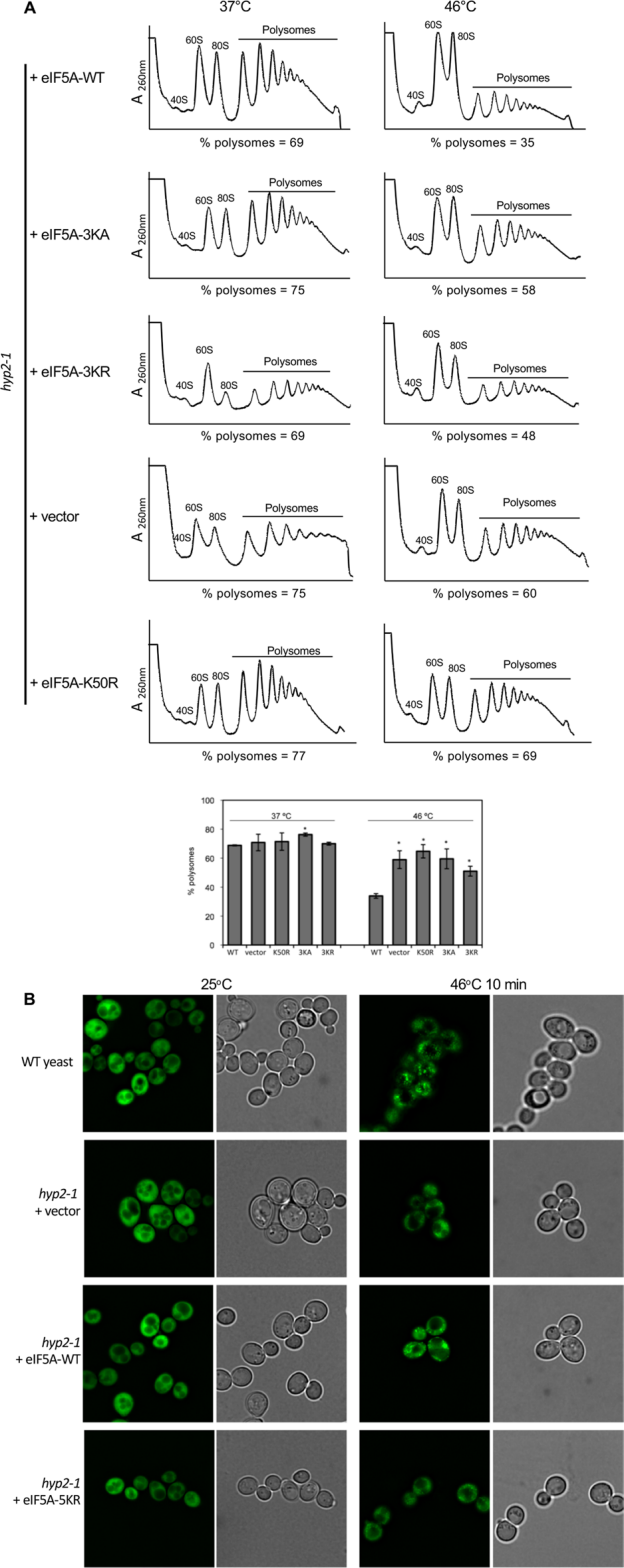
SUMOylation of eIF5A1 is important for polysome disassembly and SG formation upon heat-shock stress. **A** Yeast cells with temperature-sensitive eIF5A mutant *hyp2-1* were transformed with different alleles of Histidine- and Flag-tagged human eIF5A1 in a pAG425GPD vector or with the empty vector. Cells were grown in SC–Leu medium until early exponential phase, incubated at 37 °C for 4 h to deplete endogenous eIF5A and then subjected to severe heat shock stress (46 °C, 30 min). Representative polysome profiles after gradient fractionation of yeast extracts are shown and the ribosomal subunits (40S and 60S), monosomes (80S), and polysomes are indicated (upper panel). When translation is arrested during stress, a reduction in the intensity of polysome peaks is seen because fewer ribosomes initiate translation. At the same time, the 60S and 80S peaks increase. The percentage of polysomes from three (eIF5A WT, vector, eIF5A–K50R, eIF5A–3KA) or two (eIF5A–3KR) independent experiments is shown as the median and standard deviation (lower panel). Statistical analysis was assessed by a Student’s *t*-test. *P < 0.05. **B** *hyp2-1* yeast strains were cotransformed with the SG marker Pab1–GFP together with the Histidine- and Flag-tagged human eIF5A1 WT or 5KR or an empty vector. WT yeast transformed with Pab1–GFP was included as a positive control. The resulting strains were incubated at 25 °C or heat shocked at 46 °C for 10 min and the formation of SG was then evaluated using a fluorescence microscope

eIF5A1 is required for polysome disassembly and the formation of SGs induced by different stressors in mammalian cells [2]. Therefore, we decided to evaluate the functionality of the eIF5A1 SUMOylation mutant in yeast cells subjected to heat-shock stress. First, we analyzed the polysome profile of the temperature-sensitive eIF5A mutant *hyp2-1* transformed with different alleles of Histidine- and Flag-tagged human eIF5A1 or with the empty vector after incubation at 37 °C for 4 h to deplete endogenous eIF5A and then subjected to severe heat-shock stress (46 °C, 30 min). We observed that incubation of the cells at 46 °C reduced the polysome fraction in the cells reconstituted with eIF5A1–WT (Fig. 8A). A significant reduction was observed in those cells transformed with the empty vector or reconstituted with the hypusination mutant or with the SUMOylation mutants of eIF5A1 (Fig. 8A and Additional file 1: Fig S10), indicating that SUMOylation of eIF5A1 is important for polysome disassembly in yeast cells subjected to heat-shock stress. Finally, we evaluated the formation of SG upon heat stress in eIF5A mutant *hyp2-1* transformed with the SGs marker Pab1–GFP together with different alleles of Histidine- and Flag-tagged human eIF5A1 or with the empty vector upon heat stress. Analysis of the GFP signal revealed the formation of SGs in those cells expressing eIF5A1–WT (Fig. 8B and Additional file 1: Fig S11). However, those cells transformed with the empty vector or reconstituted with the hypusination mutant or with the SUMOylation mutants of eIF5A1 exhibited defective formation of SGs (Fig. 8B and Additional file 1: Fig S11), suggesting that SUMOylation is important for the formation of SGs induced by heat-shock stress in yeast cells.

### Mutation of the SUMOylation sites in eIF5A1 counteracts the proproliferative and promigratory effects of eIF5A1 overexpression on pancreatic ductal adenocarcinoma cells

**Fig. 9:**
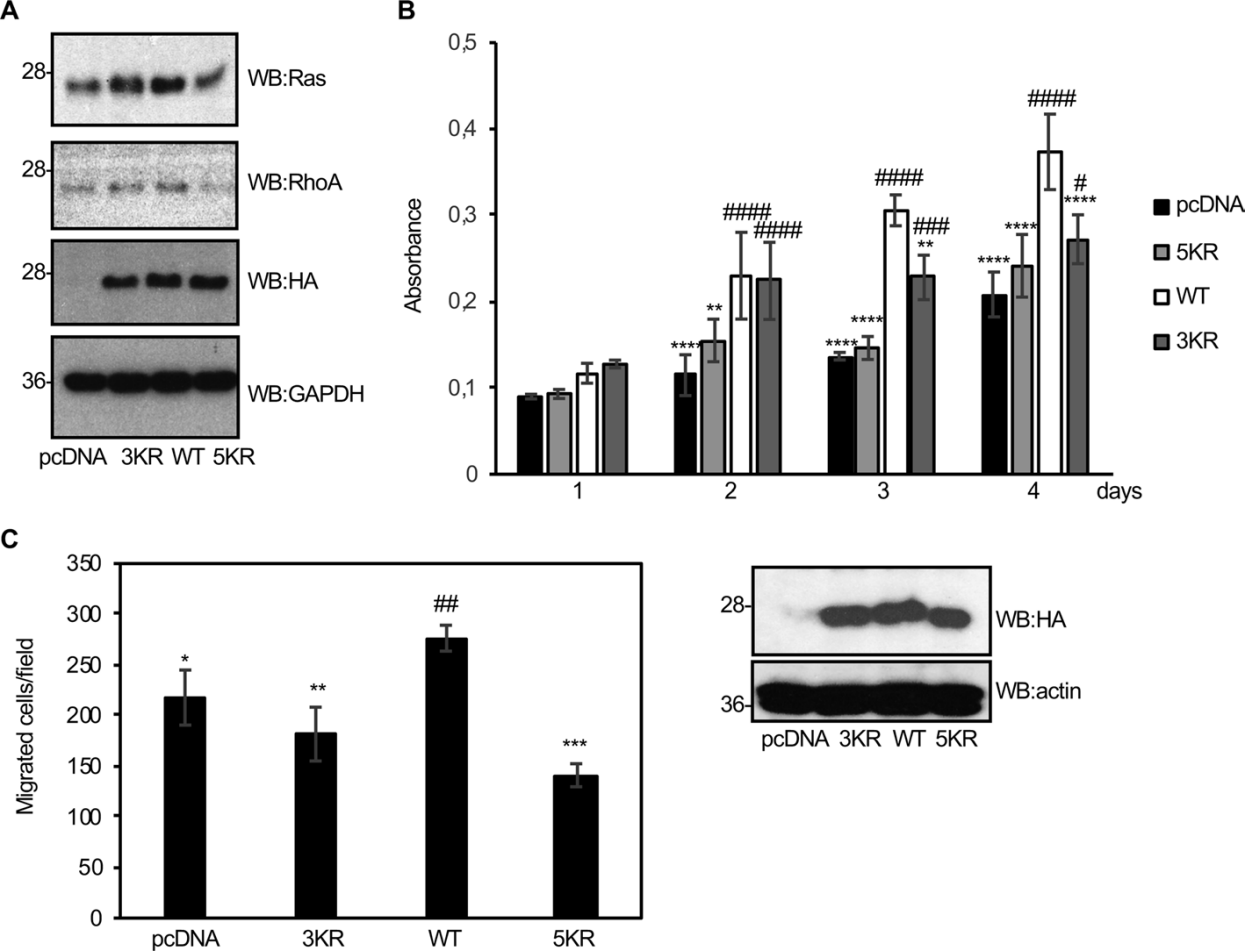
SUMOylation sites in eIF5A are essential to drive proliferation of PANC-1 cells and to promote PANC-1 cells migration in vitro. **A** Western blot analysis of RhoA and Ras in PANC-1 cells transiently transfected with HA–eIF5A1–WT, HA–eIF5A1–3KR, HA–eIF5A1–5KR, or the empty vector pcDNA. **B** PANC-1 cells stably transfected with pcDNA, HA–eIF5A1–WT, HA–eIF5A1–3KR, or HA–eIF5A1–5KR were evaluated for cell growth. Graphs show the proliferation of PANC-1 cells after the indicated times of growth. Data represent the mean and error bars of three biological replicates. ANOVA **P < 0.01, ****P < 0.0001 compared with the cells overexpressing eIF5A–WT; ###*P* < 0.001, ####*P* < 0.0001 compared with the pcDNA transfected cells. **C**, Migration of PANC1 cells stably transfected with pcDNA, HA–eIF5A1–WT, HA–eIF5A1–3KR, or HA–eIF5A1–5KR was determined by transwell migration assay (left panel). Data represent the mean and error bars of three biological replicates. ANOVA *P < 0.05, **P < 0.01, ***P < 0.001 compared with the cells overexpressing eIF5A–WT; ##*P* < 0.01 compared with the pcDNA transfected cells. Right panel shows western blot analysis of the expression of HA–eIF5A–WT or the mutant proteins in the PANC-1 cells analyzed in **B** and **C**

eIF5A is highly expressed in different types of cancer including pancreatic ductal adenocarcinoma (PDAC), in which its overexpression correlates with disease progression. eIF5A1 drives the growth and migration of PDAC cells through different mechanisms, such as the modulation of the synthesis of the cytoskeleton regulatory proteins RhoA and KRAS [48, 60–62]. Another important pathway in PDAC is the SUMO pathway [63]. Importantly, eIF5A has been identified as one of the SUMO substrates in these cells [64]. Therefore, we decided to evaluate whether mutation of the SUMOylation sites in eIF5A1 affects its ability to regulate the expression of those cytoskeleton regulatory proteins. We observed that eIF5A1–WT overexpression positively regulated the levels of both RhoA and Ras proteins in PANC-1 cells (Fig. 9A), as previously reported [48, 60]. However, transfection of the eIF5A1 SUMOylation mutants had a minor (eIF5A–3KR) or no (eIF5A–5KR) effect on the levels of both proteins compared with control cells (Fig. 9A). We then decided to evaluate whether mutation of the SUMOylation sites in eIF5A1 would alter its ability to promote PANC-1 cells proliferation and migration. Overexpression of eIF5A1–WT significantly enhanced the proliferation of PANC-1 cells (Fig. 9B), as previously reported [48]. Expression of HA–eIF5A–3KR significantly reduced the cell proliferation compared with the HA–eIF5A–WT cells (Fig. 9B) and expression of HA–eIF5A–5KR was not sufficient to drive proliferation of PANC-1 cells (Fig. 9B). Finally, we observed that overexpression of eIF5A1–WT but not the SUMOylation mutants enhanced PANC-1 cell migration in vitro (Fig. 9C). These results suggest that SUMOylation sites in eIF5A are essential for the proproliferative and promigratory activities of eIF5A1 overexpression in PDAC.

## Discussion

We report here that human eIF5A1 is modified by SUMO2 in vitro, in vivo, and under completely endogenous conditions, indicating that SUMOylation of eIF5A1 is physiologically relevant. The appearance of a unique eIF5A1–SUMO2 band in the in vitro assays suggests that only one SUMO2 molecule is conjugated to eIF5A1 at a time. In silico analysis and several proteomic data pointed to different lysine residues in eIF5A as potential SUMO acceptors [27–29, 65, 66]. Interestingly, some of these reports also suggested that the number of lysine residues susceptible to conjugate to SUMO, as well as the ratio of the protein modified by SUMO, increased upon heat stress or proteasome inhibition. Accordingly, here we show that SUMO2 can conjugate to different lysine residues in eIF5A1 and that eIF5A1 SUMOylation can be induced by stress. It has been reported that SUMO2/3 conjugates accumulate under different stress conditions [67] and that part of these conjugates correspond with SUMO2/3 chains [27] or with ubiquitin associated to SUMO2/3 conjugates [68]. Here, we observed that heat shock or proteasome inhibition strongly increase the intensity and number of eIF5A1 SUMOylated bands in cells expressing a mutant of SUMO2 unable to form chains and in the presence of an ubiquitination inhibitor, suggesting that stress induces multiple-mono-SUMOylation of eIF5A1. However, we cannot dismiss the potential involvement of endogenous SUMO or other posttranslational modifications in the emergence of the additional eIF5A–SUMO2 bands.

Importantly, we demonstrated that SUMOylation of eIF5A is conserved in yeast and that mutants of eIF5A1 in the SUMOylation sites were unable to completely complement a yeast eIF5A knockdown. The specific activities of eIF5A1 modulated by SUMO that impact yeast growth remain unidentified and will be the aim of future studies.

Our results also suggest that SUMOylation increases the stability of eIF5A by inhibiting its proteasome-mediated degradation. As it has been reported that eIF5A1 is degraded in mammalian cells via CHIP-mediated ubiquitination [21], we speculate that SUMO may negatively modulate the interaction between eIF5A1 and CHIP in these cells. Although the exact molecular mechanism by which SUMO stabilizes eIF5A is still unclear, previously reported data suggest that the stability of eIF5A may be determinant for the fate of a specific cell type upon acute heat stress [69].

eIF5A protein is mostly localized in the cell cytoplasm, but it has also been found in the nucleus, endoplasmic reticulum (ER) membrane, mitochondria, etc. eIF5A1 nuclear entry has been proposed to be mediated by a nuclear localization signal detected in the first 19 residues of eIF5A1 [70] or through passive diffusion [71], whereas exportin 4 mediates the nuclear export of eIF5A1 [72]. In addition, the nuclear/cytoplasm distribution of eIF5A1 has been reported to be dictated by an interplay between acetylation and hypusination of eIF5A1, with hypusination favoring the cytoplasmic localization of the protein [55, 73]. Here, we show that mutation of the SUMOylation sites in eIF5A1 or treatment of cells with the SUMOylation inhibitor ML-792 increases the nuclear localization of eIF5A1, suggesting that SUMOylation facilitates the localization of eIF5A1 in the cytoplasm, which could be essential for its interaction with the ribosome and for stimulating the efficient synthesis of specific subset of proteins [74–77]. Different mechanisms may mediate the regulation of the subcellular localization of eIF5A1 by SUMO. SUMO conjugation to eIF5A may difficult its passive diffusion to the nucleus or it may facilitate its interaction with exportin 4 or with other eIF5A binding partners, such as ribosomes, in the cytoplasm. In addition, a possible interplay between SUMOylation and acetylation of eIF5A1 may also contribute to modulate its subcellular localization.

How the eIF5A subcellular localization impacts on its activities is still unclear and it may depend on the cellular context. Thus, cytoplasmic eIF5A has been proposed to promote proliferation in vulvar neoplasia [78], to facilitate the translation of proteins involved in autophagosome formation in breast cancer cell lines [79], or to be involved in leiomyomas pathogenesis [80]. However, the detection of eIF5A1 in both nucleus and cytoplasm in lung adenocarcinomas [19] has led to the hypothesis of potential nuclear functions for eIF5A1.

One of the roles of eIF5A is to promote the assembly of SGs [2], cytoplasmic condensates to which it is translocated when cells are subjected to certain types of stress [81]. eIF5A is also required for the translational repression induced by stress and it has been proposed to facilitate cell survival under adverse conditions [2]. The SUMO pathway is also essential for maintaining cell homeostasis upon different stressors [82] and it is involved in SGs targeting and assembly [13–15]. Therefore, we hypothesized that eIF5A SUMOylation may play an important role in the cellular response to stress and our data suggest that SUMOylation of eIF5A is required for SGs formation and inhibition of translation after heat-shock conditions in yeast. SGs are thought to play a protective role during cellular stress, and defects in SGs formation or dissolution may contribute to the pathogenesis of different diseases including neurodegeneration and cancer [83–86], specifically pancreatic cancer [87]. The regulation of SGs formation and function by eIF5A has been suggested as a mechanism that may contribute to the protection of cancer cells from cellular stress [88].

eIF5A is upregulated in many different malignancies, such as pancreatic ductal adenocarcinoma, where a correlation between eIF5A levels and cancer progression and metastasis has been demonstrated [48, 60–62]. Our results reveal that mutation of the SUMOylation sites in eIF5A1 negatively impact the proproliferative and promigratory effect of eIF5A1 in PDAC. Different mechanisms have been proposed as mediators of the effect of eIF5A in pancreatic cancer, including facilitating the translation of a specific subset of proteins such as RhoA and Ras [48, 60]. Our data revealed that mutation of the SUMOylation sites in eIF5A1 is associated with defects in the upregulation of these proteins, suggesting that SUMOylation may facilitate the translation of a specific subset of proteins promoted by eIF5A.

Similarly to eIF5A, SUMOylation has also been proposed as a therapeutic target in PDAC [63, 64, 89, 90]. Different SUMO pathway components have been found overexpressed in PDAC [89, 90] and an association between increased SUMOylation and adverse prognostic has been reported [64, 90]. Interestingly, one of the SUMO substrates identified in PANC-1 cells was eIF5A [64], suggesting that it may be a mediator of the pathogenic effect of upregulated SUMOylation in PDAC.

## Conclusion

These results suggest that eIF5A SUMOylation plays a critical role in both the cellular response to stress and cancer pathogenesis and it may represent a target for future therapies.

### Supplementary Information

**Additional file 1: Fig. S1.** eIF5A1 protein is modified by SUMO2 *in vitro*. A *In vitro *SUMOylation assay with SUMO2 using 35Smethionine labeled *in vitro *translated eIF5A1 protein. SUMO2-modified eIF5A1 protein was then subjected to an *in vitro *deSUMOylation assay in presence of SENP1. Arrowhead indicates unconjugated eIF5A protein. Arrow indicates the SUMO2-conjugated eIF5A protein. **Fig. S2.** eIF5A1 is modified by SUMO2 in transfected cells. HEK-293 cells were co-transfected with a plasmid encoding HA-eIF5A1 and pcDNA or HA-eIF5A1, Ubc9 and His6-SUMO2. 36 h after transfection total protein extracts (WCE) and proteins fused to Histidine (His) tag were analyzed by Western-blot with the indicated antibodies. Arrowhead indicates unconjugated eIF5A1 protein. Arrow indicates the SUMO2-conjugated eIF5A1 protein. **Fig. S3.** eIF5A1 is modified by SUMO2 in cells treated with the hypusination inhibitor GC7. HEK-293 cells were cotransfected with a plasmid encoding HA-eIF5A1 together with pcDNA or Ubc9 and His6-SUMO2. 36 h after transfection, cells were treated with GC7 or DMSO. 16 h after treatment, total protein extracts (WCE) and proteins fused to Histidine (His) tag were analyzed by western blot with the indicated antibodies. Arrowhead indicates unconjugated eIF5A1 protein. Arrow indicates the SUMO2-conjugated eIF5A1 protein. **Fig. S4.** Mutation of the SUMOylation sites in eIF5A1 modulates its stability. HEK-293 cells were transfected with HAeIF5A1-WT, HA-eIF5A1-3KA, or HA-eIF5A1-5KA, and treated with cycloheximide (CHX) 24 h after transfection. Samples were collected at the indicated hours post treatment (hpt) and protein extracts were analyzed by Western-blot with the indicated antibodies (left panel). The intensity of the bands was quantified using ImageJ software. eIF5A bands intensity were normalized to GAPDH bands from each respective time and plotted (right panel). Data represent the mean and error bars of 3 biological replicates. Statistical analysis was assessed by a Student’s t-test. ***, P<0.001, compared to WT. **Fig. S5.** Mutation of the SUMOylation sites in eIF5A1 does not prevent ubiquitin conjugation. HEK-293 cells were transfected with a plasmid encoding HA-tagged eIF5A1-WT, eIF5A1-3KA or eIF5A1-5KA together with pcDNA or His6-Ubiquitin (Ub). 36 h after transfection cells were treated with MG132 for 16 h and whole-protein extracts (WCE) and Histidine (His)-tagged purified proteins were analyzed by Western-blot with the indicated antibodies. **Fig. S6.** Mutants of eIF5A1 in the SUMOylation sites are degraded by the proteasome. HEK-293 cells were transfected with HA-eIF5A1-3KA, or HA-eIF5A1-5KA, and 24 h after transfection cells were treated with MG132 or DMSO for 16h. Cells were then incubated with cycloheximide (CHX) and at the indicated times after CHX treatment, protein extracts were analyzed by Western-blot with the indicated antibodies (upper panels). The intensity of the bands was quantified using ImageJ software. eIF5A bands intensity were normalized to actin bands from each respective time and plotted (lower panels). Data represent the mean and error bars of 3 biological replicates. Statistical analysis was assessed by a Student’s t-test. *, P<0.05; ***, P<0.001. **Fig. S7.** Mutation of the SUMOylation sites in eIF5A1 modulates its subcellular localization. A A549 cells were transfected with HA-eIF5A1-WT, HA-eIF5A1-3KA, or HA-eIF5A1-5KA and 36 h after transfection localization of eIF5A was evaluated using immunofluorescence assay with anti-HA antibody. B A549 cells were transfected with HA-eIF5A1-WT or HA-eIF5A1-3KA, as indicated. 36 h after transfection cells were subjected to subcellular fractionation and the levels of eIF5A1 protein in the nucleus and cytoplasm were evaluated using Western-blot analysis with anti-HA antibody. **Fig. S8.** Analysis of poly-SUMO2 chain formation on eIF5A1 upon MG132 treatment. HEK-293 cells were co-transfected with HA-eIF5A1-WT, Ubc9 and His6-SUMO2 (S2) or His6-SUMO2-K0. 36 h after transfection cells were treated with MG132 for 16 h and whole-protein extracts and Histidine-tagged purified proteins were analyzed by Western-blot with the indicated antibodies. **Fig. S9.** An eIF5A1 SUMOylation-deficient mutant cannot substitute for yeast *HYP2*. A Histidine-tagged proteins were purified from yeast cells transformed with an empty vector, Histidine- and Flag-tagged human eIF5A1-WT or Histidine- and Flag-tagged human eIF5A1-3KA under denaturing conditions. Purified proteins were then analyzed by Western-blot using anti-Smt3 antibody. B WT and *hyp2-1 *(upper row) or *hyp2-3 *(lower row) yeast strains were transformed with different alleles of Histidine- and Flag-tagged human eIF5A1 in a pAG425GPD vector or with the empty vector. The resulting strains were streaked on synthetic complete medium lacking leucine (SC-Leu) plates, incubated at 25 °C or 37 °C for 3 days and imaged in a GelDoc documentation system. C Expression levels of the different versions of eIF5A in the indicated strains, growing at 25 °C or after 4 h at 37 °C, were analyzed by Western-blot. Ponceau staining of the membrane is shown as a loading control. **Fig. S10.** SUMOylation of eIF5A1 is necessary for polysome disassembly. Yeast cells with temperature sensitive eIF5A mutant *hyp2-1 *were transformed with Histidine- and Flag-tagged human eIF5A1-5KR mutant in a pAG425GPD vector. Cells were grown in SC-Leu media until early exponential phase, incubated at 37 ºC for 4 hours to deplete endogenous eIF5A and then subjected to severe heat shock stress (46 ºC 30 minutes). Polysomes profiles after gradient fractionation of yeast extracts are shown and the ribosomal subunits (40S and 60S), monosomes (80S) and polysomes are indicated. The percentage of polysomes is indicated. **Fig. S11.** SUMOylation of eIF5A1 is important for SG formation upon heat shock stress. *hyp2-1 *yeast strains were co-transformed with the SG marker Pab1-GFP together with an empty vector or the Histidine- and Flag-tagged human eIF5A1 WT, K50R or 3KA. The resulting strains were incubated at 25 oC or heat-shocked at 46 oC for 10 min and the formation of SG was then evaluated using a fluorescence microscope.

## Data Availability

All data generated or analysed during this study are included in this published article (and its supplementary information files).

## Abbreviations

eIF5A: Eukaryotic translation initiation factor 5A
HS: Heat shock
SGs: Stress granules
SUMO: Small ubiquitin-like modifier
