# Supplementary material for: SUMOylation modulates eIF5A activities in both yeast and pancreatic ductal adenocarcinoma cells
Source: Cell Mol Biol Lett. 2024 Jan 16;29:15. doi: 10.1186/s11658-024-00533-5 (PMC10790418; doi:10.1186/s11658-024-00533-5)
Supplement: Supplementary file 1 — Additional file 1: Fig. S1. eIF5A1 protein is modified by SUMO2 in vitro. A In vitro SUMOylation assay with SUMO2 using 35Smethionine labeled in vitro translated eIF5A1 protein. SUMO2-modified eIF5A1 protein was then subjected to an in vitro deSUMOylation assay in presence of SENP1. Arrowhead indicates unconjugated eIF5A protein. Arrow indicates the SUMO2-conjugated eIF5A protein. Fig. S2. eIF5A1 is modified by SUMO2 in transfected cells. HEK-293 cells were co-transfected with a plasmid encoding HA-eIF5A1 and pcDNA or HA-eIF5A1, Ubc9 and His6-SUMO2. 36 h after transfection total protein extracts (WCE) and proteins fused to Histidine (His) tag were analyzed by Western-blot with the indicated antibodies. Arrowhead indicates unconjugated eIF5A1 protein. Arrow indicates the SUMO2-conjugated eIF5A1 protein. Fig. S3. eIF5A1 is modified by SUMO2 in cells treated with the hypusination inhibitor GC7. HEK-293 cells were cotransfected with a plasmid encoding HA-eIF5A1 together with pcDNA or Ubc9 and His6-SUMO2. 36 h after transfection, cells were treated with GC7 or DMSO. 16 h after treatment, total protein extracts (WCE) and proteins fused to Histidine (His) tag were analyzed by western blot with the indicated antibodies. Arrowhead indicates unconjugated eIF5A1 protein. Arrow indicates the SUMO2-conjugated eIF5A1 protein. Fig. S4. Mutation of the SUMOylation sites in eIF5A1 modulates its stability. HEK-293 cells were transfected with HAeIF5A1-WT, HA-eIF5A1-3KA, or HA-eIF5A1-5KA, and treated with cycloheximide (CHX) 24 h after transfection. Samples were collected at the indicated hours post treatment (hpt) and protein extracts were analyzed by Western-blot with the indicated antibodies (left panel). The intensity of the bands was quantified using ImageJ software. eIF5A bands intensity were normalized to GAPDH bands from each respective time and plotted (right panel). Data represent the mean and error bars of 3 biological replicates. Statistical analysis was assessed by a Stude [file 11658_2024_533_MOESM1_ESM.pdf]

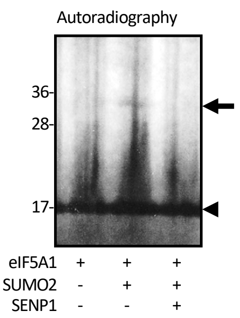

Fig. S1. eIF5A1 protein is modified by SUMO2 *in vitro*. A *In vitro* SUMOylation assay with SUMO2 using ^{35}S-methionine labeled *in vitro* translated eIF5A1 protein. SUMO2-modified eIF5A1 protein was then subjected to an *in vitro* deSUMOylation assay in presence of SENP1. Arrowhead indicates unconjugated eIF5A protein. Arrow indicates the SUMO2-conjugated eIF5A protein.

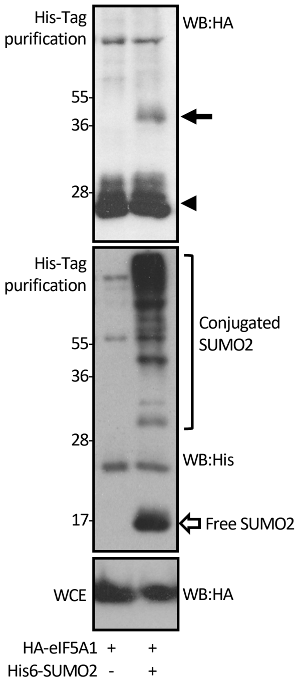

Fig. S2. eIF5A1 is modified by SUMO2 in transfected cells. HEK-293 cells were co-transfected with a plasmid encoding HA-eIF5A1 and pcDNA or HA-eIF5A1, Ubc9 and His6-SUMO2. 36 h after transfection total protein extracts (WCE) and proteins fused to Histidine (His) tag were analyzed by Western-blot with the indicated antibodies. Arrowhead indicates unconjugated eIF5A1 protein. Arrow indicates the SUMO2-conjugated eIF5A1 protein.

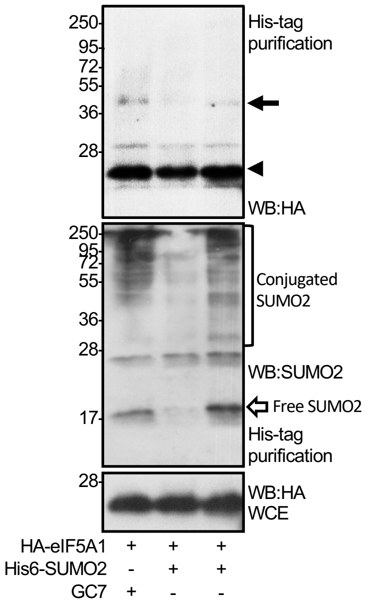

Fig. S3. eIF5A1 is modified by SUMO2 in cells treated with the hypusination inhibitor GC7. HEK-293 cells were co-transfected with a plasmid encoding HA-eIF5A1 together with pcDNA or Ubc9 and His6-SUMO2. 36 h after transfection, cells were treated with GC7 or DMSO. 16 h after treatment, total protein extracts (WCE) and proteins fused to Histidine (His) tag were analyzed by Western-blot with the indicated antibodies. Arrowhead indicates unconjugated eIF5A1 protein. Arrow indicates the SUMO2-conjugated eIF5A1 protein.

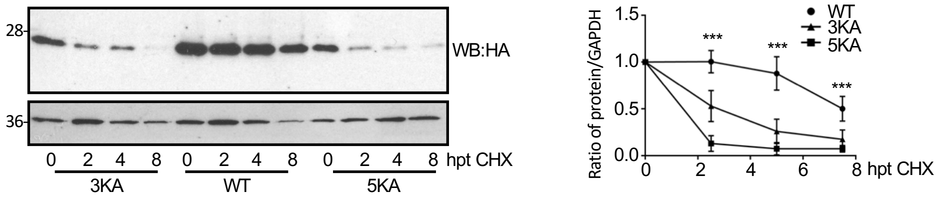

Fig. S4. Mutation of the SUMOylation sites in eIF5A1 modulates its stability. HEK-293 cells were transfected with HA-eIF5A1-WT, HA-eIF5A1-3KA, or HA-eIF5A1-5KA, and treated with cycloheximide (CHX) 24 h after transfection. Samples were collected at the indicated hours post treatment (hpt) and protein extracts were analyzed by Western-blot with the indicated antibodies (left panel). The intensity of the bands was quantified using ImageJ software. eIF5A bands intensity were normalized to GAPDH bands from each respective time and plotted (right panel). Data represent the mean and error bars of 3 biological replicates. Statistical analysis was assessed by a Student's t-test. ***, $P<0.001$, compared to WT.

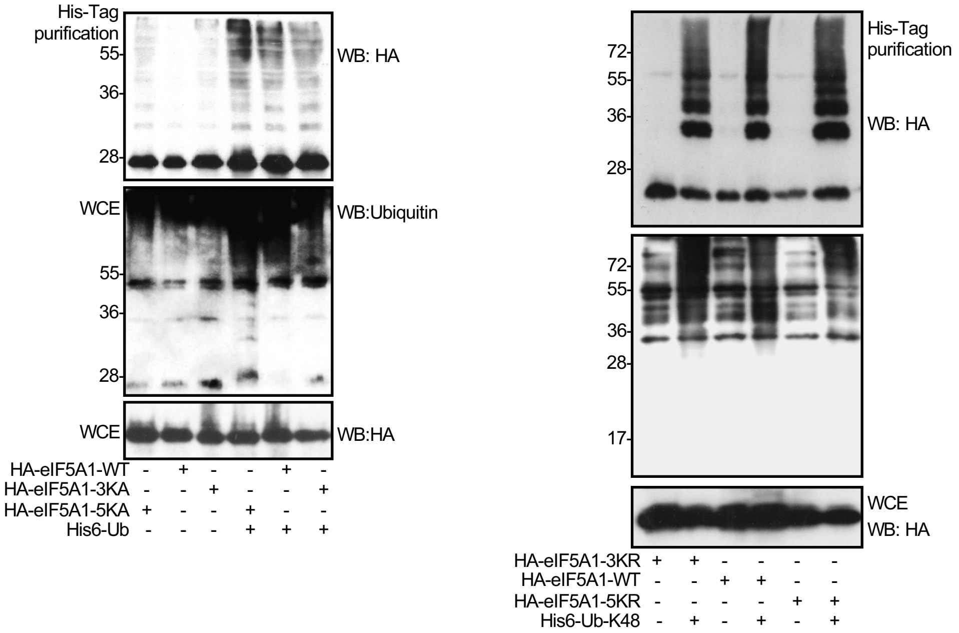

Fig. S5. Mutation of the SUMOylation sites in eIF5A1 does not prevent ubiquitin conjugation. HEK-293 cells were transfected with a plasmid encoding HA-tagged eIF5A1-WT, eIF5A1-3KA or eIF5A1-5KA together with pcDNA or His6-Ubiquitin (Ub). 36 h after transfection cells were treated with MG132 for 16 h and whole protein extracts (WCE) and Histidine (His)-tagged purified proteins were analyzed by Western-blot with the indicated antibodies.

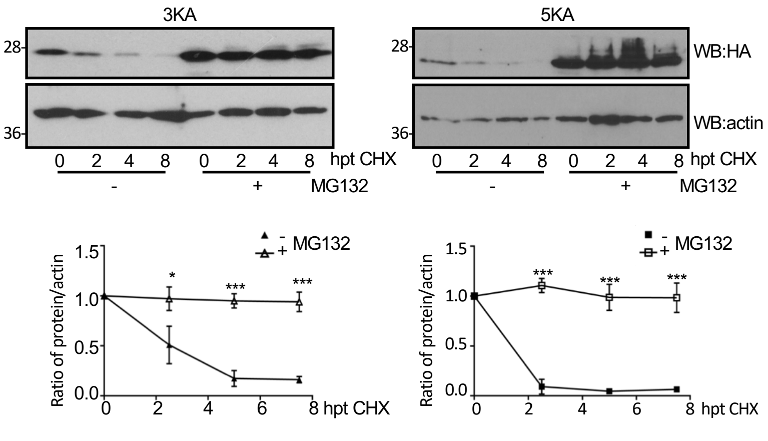

Fig. S6. Mutants of eIF5A1 in the SUMOylation sites are degraded by the proteasome. HEK-293 cells were transfected with HA-eIF5A1-3KA, or HA-eIF5A1-5KA, and 24 h after transfection cells were treated with MG132 or DMSO for 16h. Cells were then incubated with cycloheximide (CHX) and at the indicated times after CHX treatment, protein extracts were analyzed by Western-blot with the indicated antibodies (upper panels). The intensity of the bands was quantified using ImageJ software. eIF5A bands intensity were normalized to actin bands from each respective time and plotted (lower panels). Data represent the mean and error bars of 3 biological replicates. Statistical analysis was assessed by a Student's t-test. *, $P<0.05$; ***, $P<0.001$.

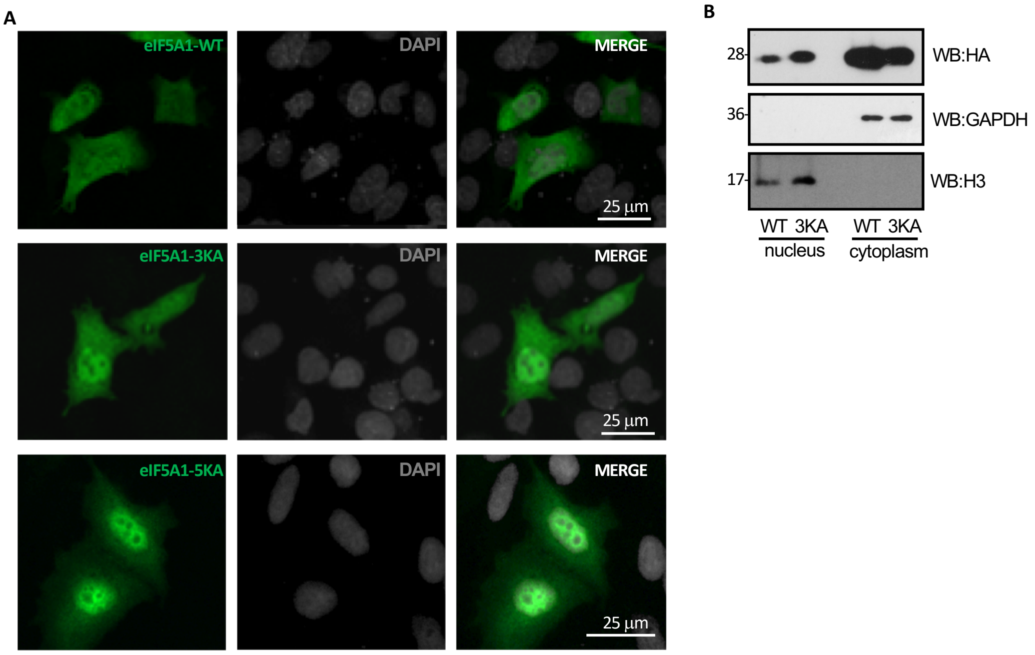

Fig. S7. Mutation of the SUMOylation sites in eIF5A1 modulates its subcellular localization. A A549 cells were transfected with HA-eIF5A1-WT, HA-eIF5A1-3KA, or HA-eIF5A1-5KA and 36 h after transfection localization of eIF5A was evaluated using immunofluorescence assay with anti-HA antibody. B A549 cells were transfected with HA-eIF5A1-WT or HA-eIF5A1-3KA, as indicated. 36 h after transfection cells were subjected to subcellular fractionation and the levels of eIF5A1 protein in the nucleus and cytoplasm were evaluated using Western-blot analysis with anti-HA antibody.

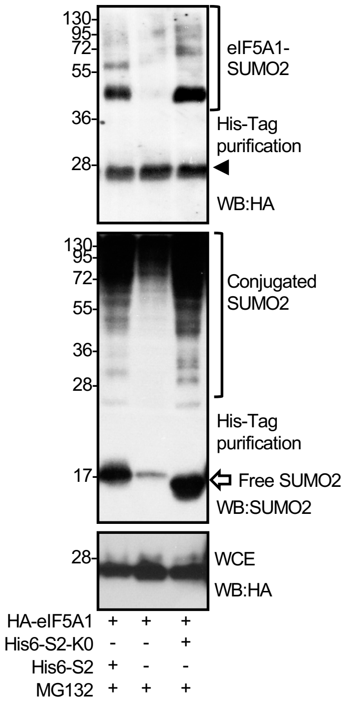

Fig. S8. Analysis of poly-SUMO2 chain formation on eIF5A1 upon MG132 treatment. HEK-293 cells were co-transfected with HA-eIF5A1-WT, Ubc9 and His6-SUMO2 (S2) or His6-SUMO2-K0. 36 h after transfection cells were treated with MG132 for 16 h and whole protein extracts and Histidine-tagged purified proteins were analyzed by Western-blot with the indicated antibodies.

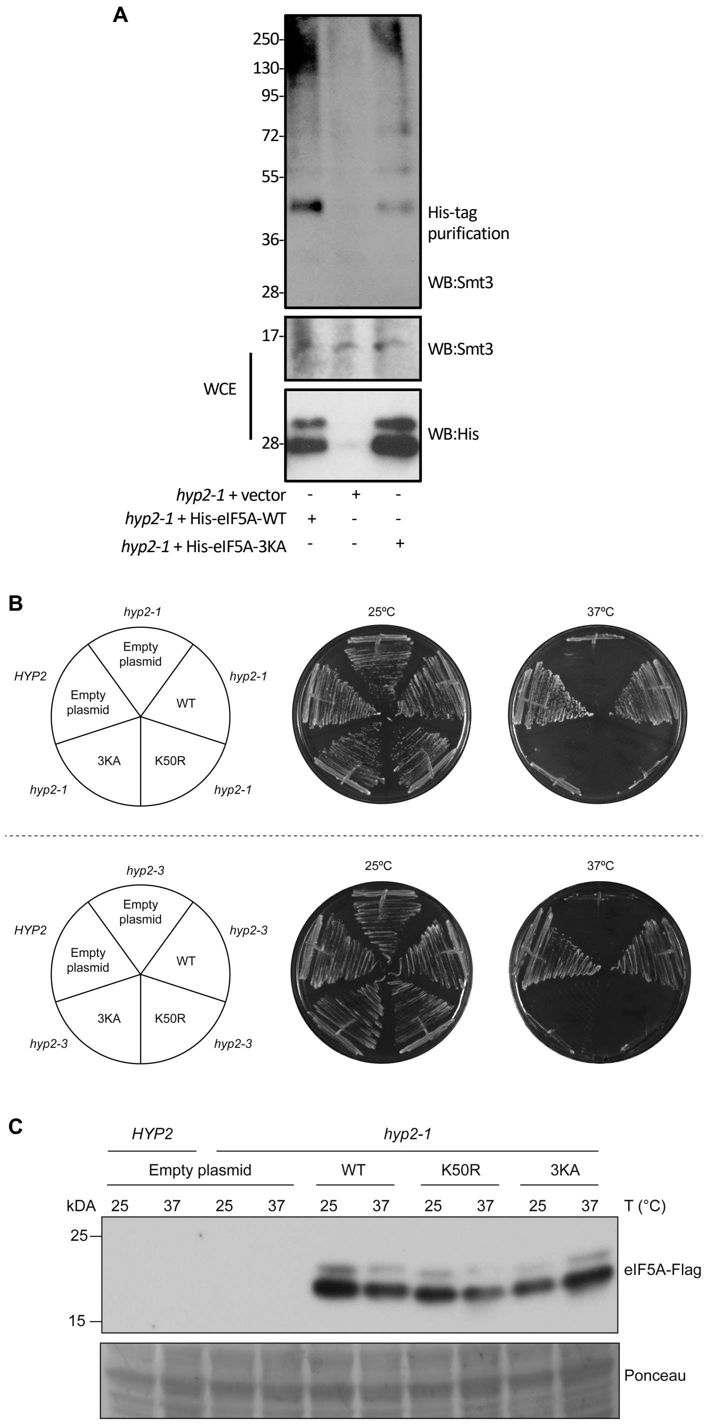

Fig. S9. An eIF5A1 SUMOylation-deficient mutant cannot substitute for yeast *HYP2*. A Histidine-tagged proteins were purified from yeast cells transformed with an empty vector, Histidine-and Flag-tagged human eIF5A1-WT or Histidine- and Flag-tagged human eIF5A1-3KA under denaturing conditions. Purified proteins were then analyzed by Western-blot using anti-Smt3 antibody. B WT and *hyp2-1* (upper row) or *hyp2-3* (lower row) yeast strains were transformed with different alleles of Histidine-and Flag-tagged human eIF5A1 in a pAG425GPD vector or with the empty vector. The resulting strains were streaked on SC-Leu plates, incubated at 25 °C or 37 °C for 3 days and imaged in a GelDoc documentation system. C Expression levels of the different versions of eIF5A in the indicated strains, growing at 25 °C or after 4 h at 37 °C, were analyzed by Western-blot. Ponceau staining of the membrane is shown as a loading control.

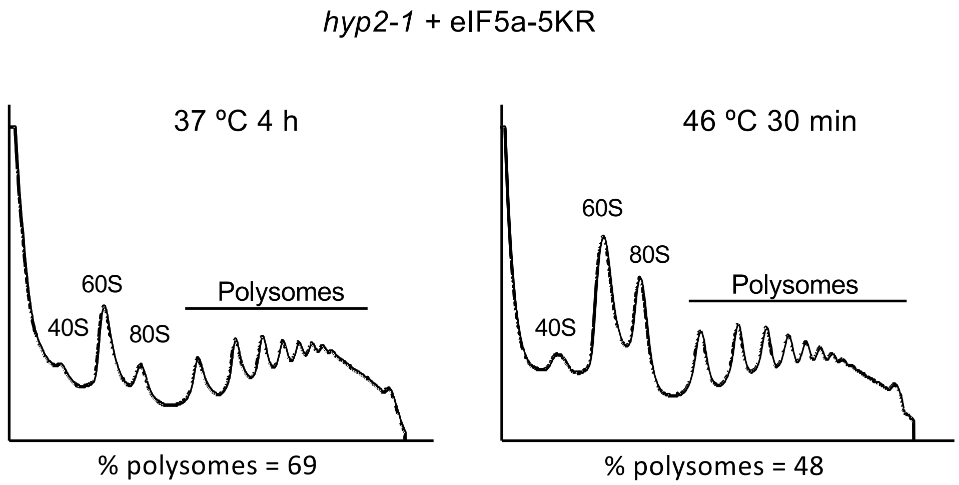

Fig. S10. SUMOylation of eIF5A1 is necessary for polysome disassembly. Yeast cells with temperature sensitive eIF5A mutant *hyp2-1* were transformed with Histidine-and Flag-tagged human eIF5A1-5KR mutant in a pAG425GPD vector. Cells were grown in SC-Leu media until early exponential phase, incubated at 37 ºC for 4 hours to deplete endogenous eIF5A and then subjected to severe heat shock stress (46 ºC 30 minutes). Polysomes profiles after gradient fractionation of yeast extracts are shown and the ribosomal subunits (40S and 60S), monosomes (80S) and polysomes are indicated. The percentage of polysomes is indicated.

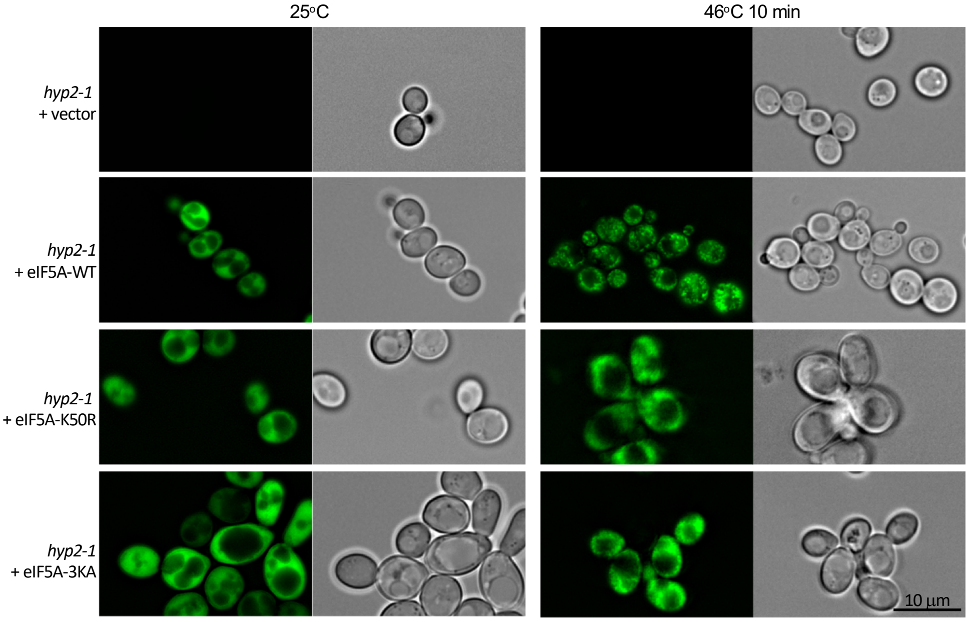

Fig. S11. SUMOylation of eIF5A1 is important for SG formation upon heat shock stress. *hyp2-1* yeast strains were co-transformed with the SG marker Pab1-GFP together with an empty vector or the Histidine-and Flag-tagged human eIF5A1 WT, K50R or 3KA. The resulting strains were incubated at 25 ºC or heat-shocked at 46 ºC for 10 min and the formation of SG was then evaluated using a fluorescence microscope.
